# Eye movements reveal a dissociation between prediction and structural processing difficulty in language comprehension

**DOI:** 10.1073/pnas.2532230123

**Published:** 2026-08-07

**Authors:** William Timkey, Kuan-Jung Huang, Byung-Doh Oh, Grusha Prasad, Suhas Arehalli, Tal Linzen, Brian Dillon

**Affiliations:** ^a^ Department of Linguistics, New York University, New York, NY 10003; ^b^ Department of Brain and Cognitive Sciences, Massachusetts Institute of Technology, Cambridge, MA 02139; ^c^ Division of Linguistics and Multilingual Studies, Nanyang Technological University, Singapore 639818, Singapore; ^d^ Department of Computer Science, Colgate University, Hamilton, NY 13346; ^e^ Department of Computer Science, Macalester College, St. Paul, MN 55105; ^f^ Center for Data Science, New York University, New York, NY 10012; ^g^ Department of Linguistics, University of Massachusetts Amherst, Amherst, MA 01003

**Keywords:** language, prediction, reading, eye-tracking, human language processing

## Abstract

In the process of extracting a meaning from a text, our eyes linger much more on some words than others, and we often reread earlier portions of the text. These disruptions to the reading process are particularly common in syntactically ambiguous sentences. What explains the difficulty presented by these sentences? One prominent hypothesis explains it as a special case of the impact of a word’s predictability (operationalized via surprisal) on the difficulty of processing the word. This contrasts with theories that attribute these disruptions to errors in the structure-building process. Earlier attempts to address this debate have been inconclusive because of small numbers of participants, coarse measurements of the reading process that are ill-suited to disentangling these competing views, and a limited range of surprisal estimates. Here, we conduct a large-scale study (n=368) examining eye movements during the reading of syntactically challenging sentences, using 409 types of surprisal estimates from language models with multiple architectures and training settings. We find a stark dissociation: Early effects of syntactic disambiguation are well-approximated by language model surprisal, but syntactic disambiguation incurs a significant additional cost, reflected in an increase in rereading that is not explained by language model surprisal. We conclude that surprisal can capture routine structure-building, but not the cost of detecting or correcting errors in the structure-building process.

## Significance

When we read sentences, our eyes move back and forth in complex ways. One factor that explains this pattern is prediction: Our experience allows us to anticipate upcoming words, making it is easier to recognize predictable ones. What are the limits on what can be explained by prediction? Here, we use contemporary language models and recordings of eye movements from hundreds of participants to answer this question. We find that language models provide only a partial explanation: Their next-word probability estimates explain the early stages of reading, but not the difficulty of integrating a word into the context, reflected in backward eye movements and rereading. This highlights the challenge of creating cognitive models that can explain all stages of reading.

Humans possess a nearly universal ability to communicate complex thoughts to one another through language. We are able to do so because languages are governed by syntactic rules, which determine how the structure of an utterance gives rise to its meaning: Although the sentences “The dog found the hiker” and “The hiker found the dog” are made up of the same words, English syntax assigns a different grammatical role to those words in each of the two sentences, based on the order of the words. We can rapidly and incrementally apply this grammatical knowledge to reconstruct the intended meaning of a sentence from speech or text (1–6). Decades of research have shown that this process is characterized by a great deal of complexity, which can lead to processing difficulty (7). In reading, processing difficulty can manifest in readers lingering on a particular word, or returning to earlier points in the sentence to reread parts of it. Such comprehension difficulty can be seen when syntactic ambiguity is resolved (2, 8, 9), at points of high syntactic complexity (10–12), when processing rare syntactic structures (13, 14), or when processing outright ungrammatical sentences (15–17).

One of the most productive approaches to study human sentence comprehension has focused on the resolution of incremental syntactic ambiguity. A sentence beginning with “The hiker found the dog...,” for example, is temporarily ambiguous between two interpretations: a more natural interpretation in which the hiker found the dog after looking for it, and a less natural interpretation in which the hiker realized something about the dog (a meaning that can be conveyed by the unambiguous “the hiker found that the dog...”). The sentence can be disambiguated in favor of the less common interpretation by a continuation such as “The hiker found the dog was a delightful companion.” While such less expected continuations are certainly allowed by the grammar of English, as soon as the sentence is disambiguated in this way, readers often experience significant processing difficulty. The effect of temporary ambiguity on processing difficulty can be isolated when the unambiguous version of the sentence is used as a control (e.g., “The hiker that the dog was a delightful companion”). A consistent finding in experiments with such sentences is that readers spend more time reading the disambiguating word (here “was”) in the temporarily ambiguous version of the sentence compared to the same word in the unambiguous control (2, 18, 19).

What cognitive processes give rise to syntactic disambiguation difficulty? Under one prominent view, readers distribute cognitive resources among many potential structural interpretations of the sentence in parallel, and shift their beliefs about the structure of the sentence as evidence accumulates (4, 20–23). Surprisal theory (4, 21) proposes that the effect of syntactic disambiguation on word-by-word processing difficulty can be fully explained by the effort required for such belief updates; this effort can be shown to be equivalent to one measure of the word’s predictability—its surprisal (i.e., its negative log probability in context).

There is extensive evidence that predictability plays an important role in language more generally: words that are more predictable from their context tend to be processed faster than less predictable ones (24), and consequently models of eye movements in reading such as E-Z Reader (25, 26) and SWIFT (27) incorporate a measure of word predictability as one of the factors that influence reading times. Given the central role of prediction in language, and the fact that one of the interpretations of the temporarily ambiguous sentence appears to be less predictable than the other, it is attractive to hypothesize, as surprisal theory does, that word surprisal on its own can account for the difficulty of syntactic disambiguation in language comprehension.

Central to any empirical test of surprisal theory are precise estimates of a word’s contextual probability. In recent years, this role has increasingly been served by neural network-based language models (LMs), machine learning systems that learn to predict upcoming words based on earlier ones. Indeed, LM-based estimates of word-by-word contextual probability have been shown to correlate with human reading measurements in short stories, novels, and news articles (23, 28, 29). These results demonstrate the importance of surprisal in language comprehension, but they do not yet speak to the hypothesis that surprisal can account for the full magnitude of processing difficulty in syntactically challenging sentences—a magnitude that can sometimes be quite substantial—because the naturalistic texts that participants read in these studies are not explicitly designed to probe the different types of structural processing difficulty, and therefore the critical sentences are not well-represented in these studies (30).

A number of recent studies have responded to this challenge with targeted tests of LM surprisal’s ability to predict processing difficulty caused by syntactic disambiguation in carefully selected sentences. These studies have found that LM surprisal successfully predicts the existence of some amount of processing difficulty at such disambiguation points (30–33), but it dramatically underpredicts the extent of the slowdown associated with such disambiguation, sometimes by orders of magnitude (30, 33–36). This raises the possibility that surprisal only explains a subset of the processes involved in syntactic comprehension, a possibility supported by successful computational models of eye movements in reading that decouple predictability effects from underlying structural processing (25, 37, 38), and by studies of naturalistic reading indicating that different indices of structural processing are useful predictors of reading time measures over and above surprisal (39–41). Overall, while surprisal theory has made a range of successful predictions, there are growing indications that it fails to provide a complete account of central aspects of structural processing (24).

While prior work certainly lends credibility to the hypothesis that surprisal only plays a partial role in syntactic disambiguation, a conclusive test of the hypothesis is yet to be conducted. As mentioned above, studies where participants read natural texts provide limited information on syntactically challenging contexts, as those contexts are generally infrequent. And prior targeted studies of the gap between LM surprisal’s predictions and human reading times in the critical syntactic structures (30, 33, 36) have used coarse behavioral measures, primarily the latency of button presses in paradigms where the sentence is revealed word-by-word when the participant presses a button (self-paced reading, 42); these paradigms are not only unnatural, but conflate multiple distinct stages of processing into a single measure and complicate the interpretation of the findings.

Here, we use the much more sensitive eye-tracking-while-reading paradigm (43): We present participants with an entire sentence at once, and record their eye movements as they read it naturally. This detailed eye movement record makes it possible to derive multiple measures of difficulty associated with different processing stages (26, 44, 45). In particular, we can distinguish the amount of time readers spend on a word during their initial left-to-right pass—a stage that is highly sensitive to the frequency and contextual predictability of the word (24–26, 46, 47)—from later processes reflected in regressive eye movements to an earlier point in the sentence that are followed by rereading of certain parts of the sentence (2, 26, 48, 49).

We collect eye movement recordings from 368 participants who read a diverse set of syntactically challenging constructions (the materials are drawn from ref. 35, a study which used the simpler self-paced reading methodology). We then use this dataset to evaluate the predictions made by 409 different LM-based surprisal estimators, spanning a diverse range of LM architectures, for four different measures derived from the eye movement record. We find a clear dissociation between early and late reading measures: LM surprisal is largely able to explain the direction and magnitude of the processing difficulty associated with syntactic disambiguation in cases where the eyes move forward from the disambiguating word to the next word; but it fails to explain measures associated with regressions and rereading, measures that are plausibly associated with the additional costs that result from failure to integrate the disambiguating word with its context.

One possible explanation for the gap between the predictions of LM surprisal and empirical human reading times identified in prior work is that surprisal estimates from the handful of LMs used in previous studies are not well-calibrated to humans’ probabilistic syntactic knowledge, and consequently a more human-like probability model may be able to fully capture the magnitude of syntactic processing effects (28, 50). Our findings challenge this hypothesis. The success of LM surprisal in predicting forward eye movements suggests that LMs’ expectations are plausibly well-calibrated to those of humans, at least in the English LMs tested here. At the same time, the consistent failure of LM surprisal to predict later measures, across all 409 models tested, suggests it is unlikely that this misalignment reflects the idiosyncrasies of a particular LM. Overall, we find that the patterns of human-model alignment and misalignment are surprisingly consistent across all models tested.

This study thus establishes a clear empirical dissociation. In trials where the reader continues forward in the sentence from the critical word, the difficulty associated with overcoming syntactic ambiguity can be predicted by LM surprisal. But surprisal cannot explain the substantial processing cost that arises in syntactic disambiguation when readers move backward and reread parts of the sentence, cases that we hypothesize reflect error-driven reanalysis of the structure of the sentence. This pattern of results supports a role for word surprisal in syntactic disambiguation, but speaks against the stronger hypothesis that surprisal can fully explain it. Rather, our findings favor a view that integrates surprisal into a multistage account of processing difficulty.

## Measuring Processing Difficulty from Eye Movements

When we read, our eyes do not move smoothly from the start to the end of each line. Instead, they move through a text in a series of fixations, where the eyes remain stably focused on a single target point, and saccades, where the eyes rapidly move from one fixation target to another. Processing difficulty is typically linked to an increase in the duration of fixations on the current word, and an increase in the rate at which saccades leaving the current word move backward to a preceding word (regressive interword saccades), instead of forward to the following word. In this work, we focus only on saccades between words (interword saccades) and not saccades within a single word (intraword saccades).

**Fig. 1.**
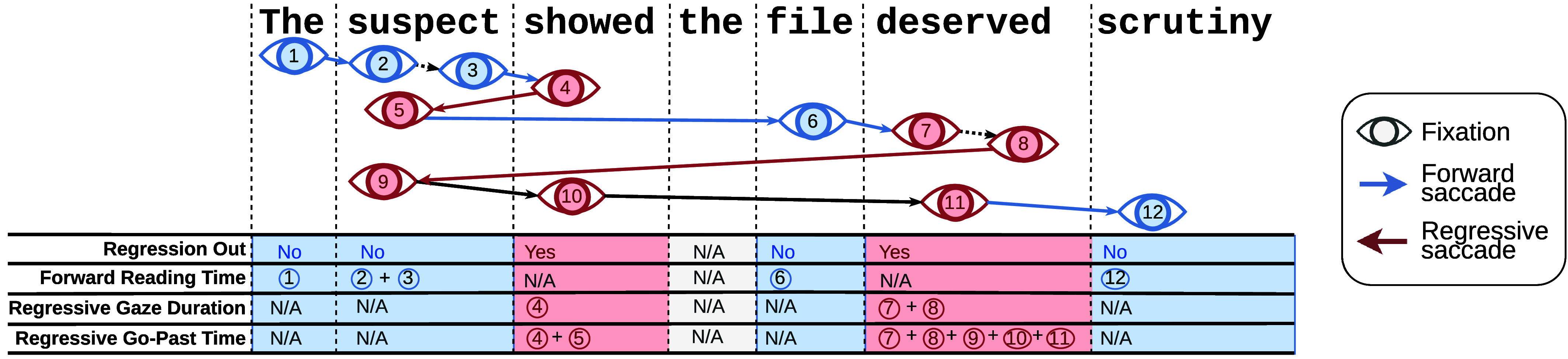
A visualization of how each word-level eye-tracking measure is computed from a sequence of fixations in a single trial. The color of fixations corresponds to the direction of the saccade that exits the word. All other saccades (non-first-pass and intraword) are colored black.

We select four reading measures in this work. Unlike some other measures used in the eye-tracking literature, all four measures only refer to the current word ($w_{i}$) and previous words ($w_{<i}$); this makes it possible to relate these measures to the surprisal of $w_{i}$, which naturally cannot take into account the words that follow $w_{i}$. These definitions all make reference to first pass fixations—any fixations on $w_{i}$ from when it is fixated for the first time before a saccade exits this word, but only in the first pass through the word; that is, given that all prior fixations were only on $w_{i}$ or words to its left $w_{<i}$. The four measures are as follows (see Fig. 1 for a graphic illustration):

1. **Forward Reading Time**: The total duration of all first-pass fixations on word $w_{i}$ in trials where the saccade which exits $w_{i}$ is a forward saccade to $w_{>i}$ (i.e., to the right); other trials are excluded when computing this measure. This measure is typically taken to capture the cost of processing $w_{i}$ when the process of integrating it into its context proceeds without errors (25–27).
2. **(First-Pass) Regressions Out**: The proportion of saccades out of $w_{i}$ that were regressive (i.e., to the left) out of all saccades made after the first-pass fixations on $w_{i}$. Models of eye movements in reading associate regressions with difficulty and errors related to integrating $w_{i}$ with its context (26, 27).
3. **Regressive Gaze Duration**: The total duration of all first-pass fixations on word $w_{i}$ in trials where the saccade that exits $w_{i}$ is a regressive saccade (i.e., to the left). Models of eye movements in reading associate this measure with the partial cost of processing $w_{i}$ before an error-driven regressive saccade is executed (26).
4. **Regressive Go-Past Time**: This measure, computed only for trials where the reader’s first pass at $w_{i}$ ends in a regressive saccade, is the sum of the duration of all first-pass fixations on word $w_{i}$, plus the duration of all subsequent fixations on the preceding words $w_{\leq i}$ until any word $w_{>i}$ is fixated. This measure is generally taken to reflect the cost of integrating $w_{i}$ with its context when this process is interrupted or runs into errors (44, 51).

These four measures can be distinguished by their time course: Forward Reading Time and Regressive Gaze Duration are the earliest of the four measures, as they are only sensitive to the first-pass fixations on a word; Regressions Out is a later measure, as it indexes information about the saccade that follows the first-pass fixations; and Regressive Go-Past is the latest of the four, as it additionally includes fixations after the reader regresses from the word.

The fixation time measures we analyze are regression-contingent (52–54): Their definitions depend on the direction (forward or regressive) of the saccade that exits the word after the first-pass fixations on the word. This contrasts with more typical eye-tracking-while-reading measures, where fixation times are aggregated over both exit saccade directions. Our decision to analyze regression-contingent fixation measures has several empirical and theoretical motivations discussed in *SI Appendix*, section 2; we present an analysis of more standard aggregate measures in *SI Appendix*, section 2A. The aggregate and regression-contingent analyses generally converge in their findings, but, as shown in the Appendix, many of the insights afforded by the regression-contingent analysis are obscured in the aggregate analysis. In this work, we refer collectively to Regressive Gaze, Regressive Go-Past, and Regressions Out as regressive reading measures, because all three index processing associated with regressive eye movements, in contrast with Forward Reading Time.

## LM Surprisal Explains Forward Reading but Not Rereading

### Data Collection and Effects of Interest.

**Fig. 2.**
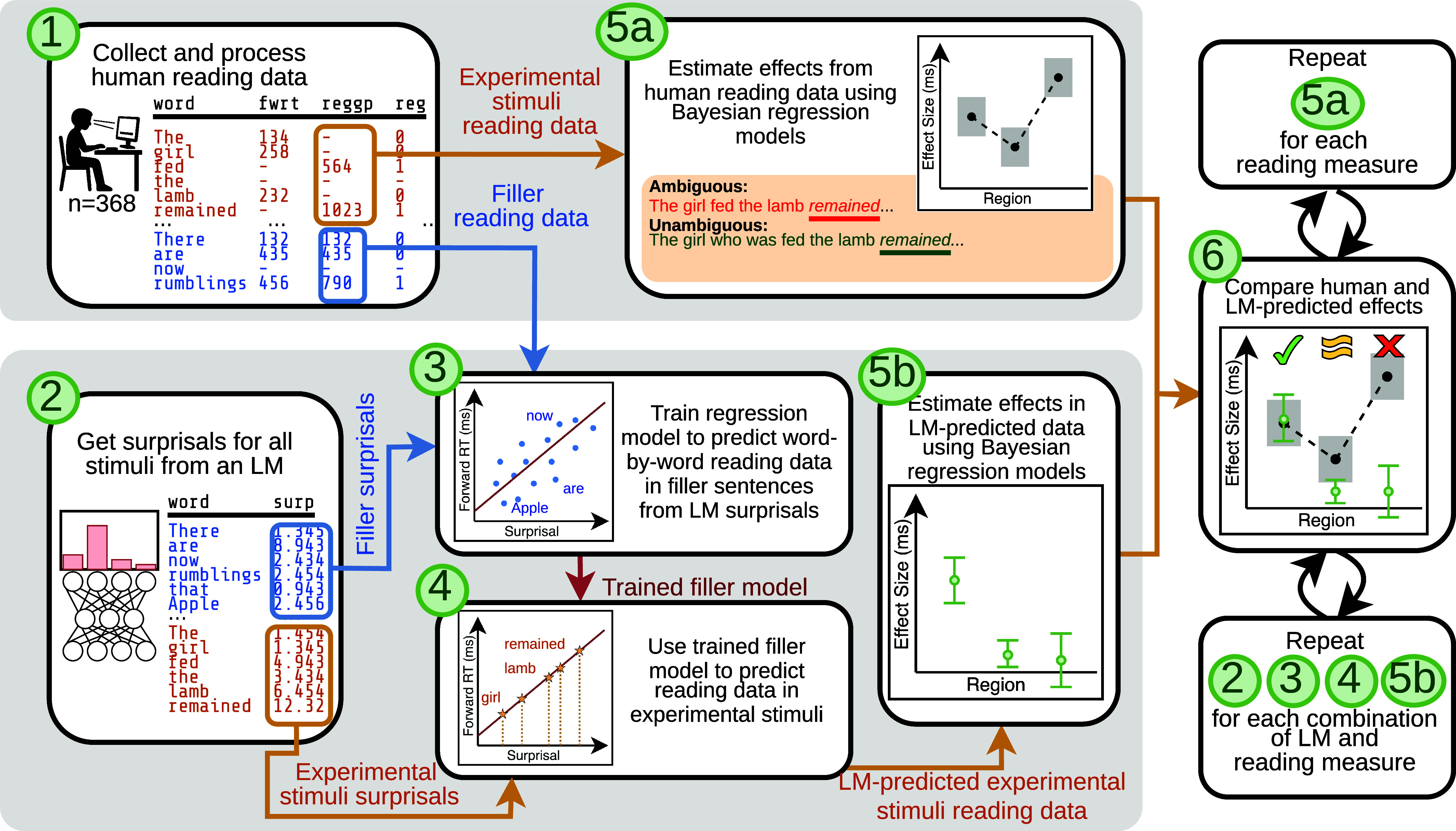
An overview of the methods employed in this work. The empirical effects due to syntactic disambiguation were first estimated from eye-tracking data (*Top*), which were then compared against those predicted by LM surprisal (*Bottom*).

We analyze eye movement data from 368 participants (out of a total of 424 participants who completed the study; the remainder are excluded based on the criteria described in *Materials and Methods*). Participants read sentences from six types of syntactically challenging constructions that have been key to the development of psycholinguistic theories; each syntactically challenging sentence is matched with a control sentence that is maximally similar to it while avoiding the syntactic challenge in question (Fig. 2, step 1; see *Materials and Methods* for the list of constructions and example sentences). These syntactically challenging (“experimental”) and control sentences are interspersed with naturalistic filler sentences sampled from a corpus of news text and novels (55). Five of the six types of syntactically challenging constructions contain a particular word where processing difficulty is predicted to arise and manifest in readers’ eye movements; we refer to this word as the critical word. The sixth construction compares processing difficulty in object and subject relative clauses, and has three different words where difficulty is predicted to differ between the conditions: the determiner, the noun, and the verb.

We calculate the empirical effects of interest as the difference in reading time or regression rates between the experimental and control sentences in each construction (Fig. 2, step 5a). We measure this difference separately for the critical word and the spillover word, that is, the word that immediately follows the critical word. We do so because processing difficulty on word $w_{i}$ is often reflected in reading behavior at $w_{i+1}$, a phenomenon referred to as spillover (56, 57). For the relative clauses, we estimate effects at the determiner, noun, and verb regions. We estimate the effect sizes in each of the four reading measures using Bayesian regression models (*SI Appendix*, section 7A).

The clearest patterns of empirical results across multiple eye movement measures—and therefore the best testbeds for the surprisal hypothesis—emerge in two sets of constructions: The relative clauses and the three constructions where the beginning of the sentence has a preferred structural parse that then turns out to be inconsistent with the full sentence. We refer to these three constructions—Main Verb/Reduced Relative (MV/RR), Intransitive/Transitive (NP/Z), and Noun Phrase/Sentential Complement (NP/S)—as the “classic garden path constructions,” following the traditional terminology in psycholinguistics (2, 58). We primarily focus on these two sets of constructions, but provide results for the remaining two constructions, subject-verb agreement violations and attachment ambiguities in *SI Appendix*, section 4.

### Predicting Reading Behavior from LM Surprisal.

We follow the filler-to-critical generalization paradigm used in previous studies (30, 34, 35); this paradigm is illustrated as steps 2 to 6 in the lower half of Fig. 2. We first obtain LM surprisal estimates for each of the words in our filler and experimental stimuli (Fig. 2, step 2). We obtain 409 sets of estimates derived from LMs from six families. We evaluate a diverse range of LMs to maximize our chances of identifying an LM that can explain the full magnitude of syntactic disambiguation difficulty. These include both standard LMs and LMs with memory constraints inspired by humans. The standard LMs are as follows (for full details, see *SI Appendix*, section 1): the Pythia suite (59) of eight transformer (60) LMs of various sizes from 70 million to 12 billion parameters, with nineteen snapshots of each model taken over the course of training, for a total of 152 LM instances; the GPT-2 family (61) of four transformers, each varying in parameter count; the Mamba family of four state-space models (62), each varying in parameter count; a family of 125 Long Short-Term Memory (LSTM; 63) language models (64) spanning a variety of random initializations, training corpora, and model sizes, which we refer to as LSTM (vS); and another LSTM language model (65), which we refer to as LSTM (G).

We further test two LM families that incorporate hypothesized constraints on human working memory. The first is a family of 10 Recurrent Neural Network Grammars (RNNGs; 66, 67) that differ in the number of distinct syntactic analyses they can maintain in parallel (ranging from one to 1,000). The RNNGs are unique among the LMs we test in that they are trained with explicit representations of the syntactic structure of the sentence. Second are two resource rational lossy-context surprisal LMs, which implement a version of surprisal that aims to take human memory limitations into account (68, 69). In these lossy-context models, context tokens are forgotten with some probability, and next-word predictions are made based on this lossy representation.

For each combination of LM instance and reading measure, we fit a regression model that predicts word-level reading measurements on filler sentences for each participant from the LM’s surprisal estimates as well as three baseline predictors: word frequency, word length, and word position (Fig. 2, step 3). Each observation in these regression models is a single reading measurement at a single word from a single trial. These regression models, which we refer to as filler models, are crucially fit only to reading data from the filler sentences, not the experimental sentences. For each measure, we also fit a baseline regression model, which predicts the same data from the three baseline predictors only. We use linear regression for the continuous measures and logistic regression for Regressions Out; see *SI Appendix*, section 7A for details.

We then generate predicted reading measurements from the 409 filler models at the critical and spillover regions in the held-out experimental sentences (Fig. 2, step 4). By maintaining this test-train split, we test a key prediction of surprisal theory: If surprisal can fully explain processing difficulty in complex and simple sentences alike under a single mechanism, then the relationship between surprisal and processing difficulty in the filler sentences should generalize to the syntactically challenging experimental sentences (30). From the LM-predicted data, we derive predicted effects using regression models analogous to those used to analyze the empirical data. Both the human and LM-predicted effect estimates take participant-level and item-level uncertainty into account. Due to computational resource limitations, we fit Bayesian regression models to LM-predicted reading data only from the one LM instance in each of the six LM families whose filler models achieved the highest per-observation goodness-of-fit averaged across all four measures: Following the logic of the generalization paradigm, we expect the LM which best explains filler data to also best explain the experimental data. For the remaining LMs, we fit frequentist regression models with identical specifications to the Bayesian models. Results from all 409 models are presented in *SI Appendix*, Fig. S4.

**Fig. 3.**
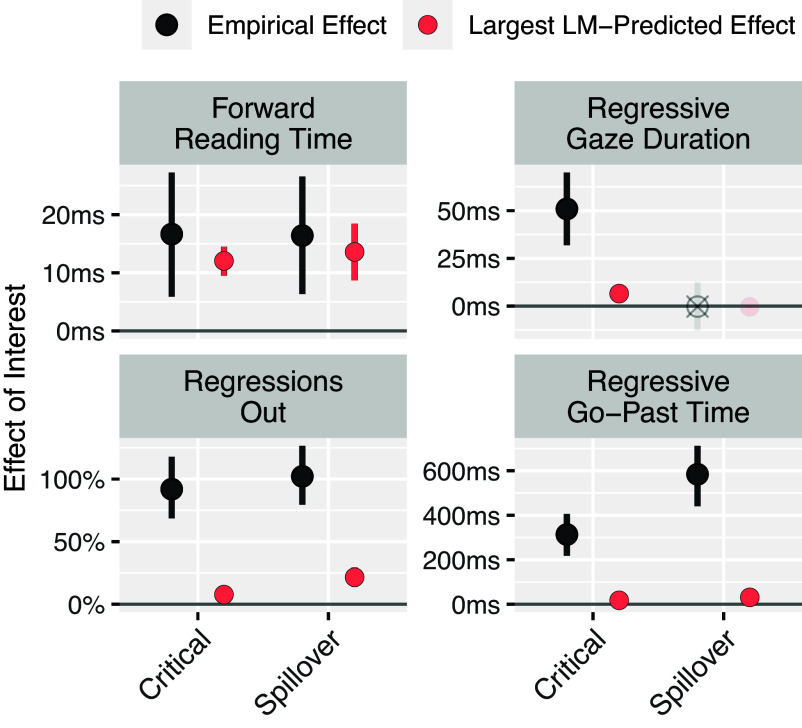
Effect estimates averaged across the three constructions in the classic garden path subset (MV/RR, NP/Z, NP/S) for both empirical (human) reading data and the LM which predicted the largest effect at each region. For reading times, the effect size is the difference in average reading time between the critical words in the ambiguous condition and those words in the unambiguous condition. The effect size in Regressions Out is the ratio of regressions in the ambiguous condition to regressions in the unambiguous condition. Error bars denote 95% credible intervals as estimated by the Bayesian regression models.

**Fig. 4.**
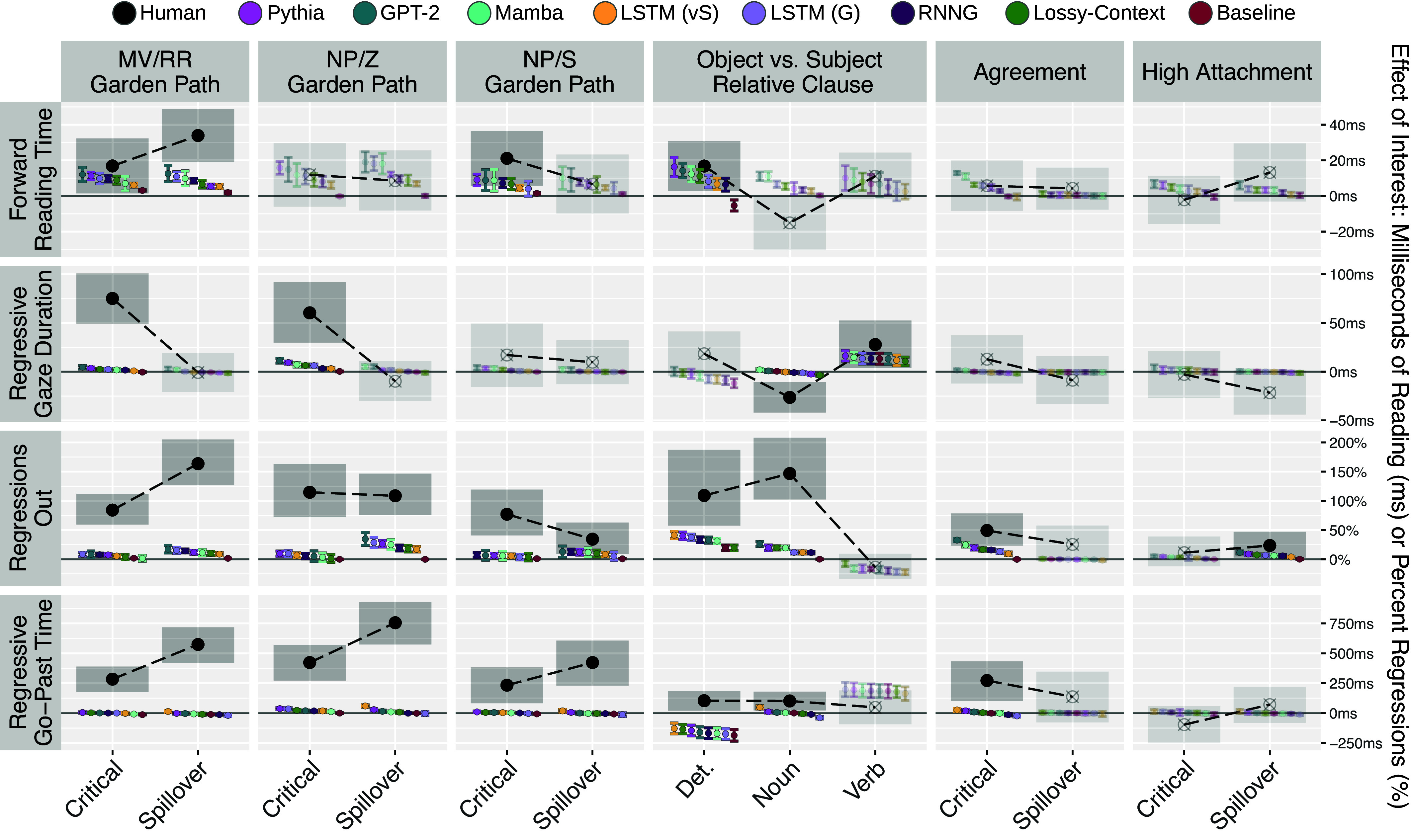
Empirical (human) and predicted (LM) effects of interest at the regions of interest in four selected constructions (columns) and four measures (rows). Human effect estimates are represented with black points for the mean, with shaded regions representing 95% credible intervals in the vertical dimension. Empirical null effects are denoted with lighter shading and an X on the mean estimate point. Colored points are predicted effect sizes from the LM within each architecture whose surprisal values provided the best fit to reading data in the filler sentences, measured by filler model log-likelihood. Within each plot, predicted LM effects are sorted from largest to smallest, with error bars denoting 95% credible intervals, incorporating participant-level and item-level uncertainty.

Finally, we compare the LM-predicted effect estimates with the empirical effects (Fig. 2, step 6). To claim that LM surprisal captures an experimental effect, the magnitude of the effect predicted by LM surprisal should match the magnitude of the effect observed in human reading. We plot the largest LM-predicted effects against the empirical effects in the garden path constructions in Fig. 3, and in all constructions for the selected LM instance from each LM family in Fig. 4. Results for all 409 LMs are plotted in *SI Appendix*, Fig. S4. Alongside visual comparisons, we quantify the evidence for and against the equality of empirical and LM-predicted effect sizes through a Bayes factor analysis (70). Detailed methods and results from this analysis are presented in *SI Appendix*, section 5.

### LM Surprisal Can Explain FORWARD READING TIME in Syntactically Challenging Sentences.

There is substantial overlap between the predictions of LM surprisal and the empirical Forward Reading Time measurements. When we aggregate the experimental results across the three classic garden path constructions (MV/RR, NP/S, and NP/Z), we find an empirical disambiguation effect at both the critical (17 ms; 95% credible interval [6 to 27 ms]) and spillover word (16 ms [6 to 26 ms]); see Fig. 3. LM surprisal from multiple LM families is able to predict the full magnitude of these effects: Closest to the human effect magnitudes is GPT-2, which predicts an aggregate garden path effect of 12 ms [7 to 18 ms] at the critical word and 12 ms [8 to 16 ms] at the spillover word. Likewise, in the relative clause construction, Forward Reading Time in the challenging condition (ORC) is 17 ms [3 to 31 ms] greater than the control condition (SRC) at the determiner region; this is consistent with GPT-2 surprisal’s predicted effect of 14 ms [11 to 18 ms]. The only exception where surprisal underpredicts Forward Reading is at the spillover word in the MV/RR construction, where the empirical effect of 33 ms [19 to 48 ms] is three times larger than the GPT-2 prediction of 11 ms [7 to 15 ms]. The Bayes factor analyses were consistent with the qualitative assessment based on visual inspection: Across a range of plausible priors, we find moderate to strong evidence in favor of the hypothesis that surprisal is sufficient to capture the full cost of syntactic disambiguation in forward reading time (*SI Appendix*, section 5).

### LM Surprisal Fails to Explain Rereading in Syntactically Challenging Sentences.

We now turn to the measures associated with rereading: Regressions Out, Regressive Gaze, and Regressive Go-Past. When we aggregate the three classic garden path constructions, the overall rate of regressions is 92% [69 to 118%] higher in the ambiguous sentences than the unambiguous controls at the critical word, and 102% [79 to 126%] higher at the spillover word (Fig. 3). This is many times larger than the largest LM-predicted effects of 8% [5 to 10%] at the critical word (from Pythia) and 21% [14 to 29%] at the spillover word (from GPT-2). A similar pattern of results holds for the regressive reading time measures. The mean reading time preceding a regression (Regressive Gaze) is 51 ms [32 to 70 ms] longer in ambiguous sentences than unambiguous control sentences. By contrast, the largest LM-predicted effect in Regressive Gaze is 6 ms [5 to 8 ms] from GPT-2, nearly eight times smaller than the human effect. The most drastic misalignment between human reading and LM predictions is in Regressive Go-Past, where we observe an empirical effect of 313 ms [218 to 406 ms] at the critical word and 584 ms [441 to 712 ms] at the spillover word. By contrast, the largest LM-predicted effect is eleven times smaller at the critical word (29 ms [18 to 40 ms] from the RNNG) and 20 times smaller at the spillover word (29 ms [20 to 37 ms] from LSTM (vS)). Three of the model families—LSTM (G), RNNG, and Mamba—fail to predict not only the magnitude, but also the direction of the Regressive Go-Past effect at the spillover word.

In the relative clause subset, LMs likewise fail to predict effect magnitudes in the measures associated with rereading. Humans show a 109% [58 to 187%] increase in regressions at the determiner in the object relative clause condition over the subject relative clause condition; this contrasts with the largest LM-predicted effect of 36% [29 to 43%] from Pythia. The mismatch is even more pronounced at the noun, where we observe a 154% [108 to 216%] increase at the noun compared to the largest LM-predicted effect of 24% [19 to 29%] from GPT-2. In contrast to the empirical pattern at the determiner and noun, there is a small decrease in regressions in the ORC condition at the verb of 23% [2 to 42%]. While at this word the human and LM-predicted effects overlap, this is also the case for the baseline filler regression model that does not include LM surprisal, indicating that LM surprisal is not necessary to predict this effect.

Empirical effects in Regressive Go-Past are much smaller in the relative clause subset than the classic garden path subset. At the determiner, all LM-predicted effects in Regressive Go-Past are in the opposite direction of those observed in humans. There is also an effect of 102 ms [23 to 180 ms] at the noun, which overlaps with the prediction of 37 ms [22 to 52 ms] from only one model, LSTM (vS).

In summary, the misalignment between the empirical and LM-predicted results is particularly clear in regressive measures in the three classic garden path constructions, where LM surprisal underpredicts both the frequency (Regressions Out) and duration (Regressive Go-Past Time) of rereading by an order of magnitude. In the relative clause subset, the misalignment is clearer in Regressions Out than the other regressive measures.

### Discussion.

Our empirical data reveal syntactic disambiguation effects in both the relative clauses and classic garden path constructions, in all of the reading measures we analyzed. LM surprisal predicts similar effect magnitudes to those observed in humans in Forward Reading Time, but consistently fails to predict the rate at which readers regress (Regressions Out), or the amount of time they spend rereading when encountering the critical word of a syntactically challenging sentence (Regressive Go-Past).

The similarity of the human and LM-predicted effects in forward reading is not simply due to the fact that empirical effect magnitudes are generally smaller in Forward Reading Time than in the rereading measures; in fact, LM surprisal often predicts larger effect magnitudes in Forward Reading Time than in Regressive Go-Past or Regressive Gaze, the opposite of the human pattern. This reflects the fact that the surprisal coefficients in the filler models are larger in Forward Reading Time than Regressive Gaze, and were similar in magnitude to those in Regressive Go-Past (*SI Appendix*, section 3A). This is likely driven by the fact that LM surprisal accounts for a greater proportion of variance and a larger absolute slowdown per bit of surprisal in forward reading time than it does in other measures (*SI Appendix*, section 3B).

Could surprisal from stronger LMs, with higher next-word prediction accuracy, better predict human rereading behavior? As we show in *SI Appendix*, section 9, the answer to this question is no: In fact, LMs with more parameters and more training data—two properties that are strongly associated with better next-word-prediction accuracy (59)—consistently predicted smaller garden path effects and consequently poorer alignment with humans; this finding parallels similar findings from an analysis of naturalistic reading (29). We also find that misalignments between humans and LMs in the regressive measures were not alleviated by imparting models with additional memory limitations: Results from the memory-limited lossy-context models were not qualitatively different from those of LMs without such limitations. Finally, there was little difference in the magnitude of predicted effects between the RNNG models, which explicitly model syntactic structure, and the other LM families, which do not, and misalignment persisted regardless of the number of parses the RNNG models could maintain in parallel. In summary, the inability of LM surprisal to predict the full costs of syntactic processing is unlikely to be remedied solely by making LMs larger and more powerful, by endowing them with explicit syntactic representations (in line with the findings of 34), or through existing approaches to imparting memory constraints.

More broadly, capturing the full effect magnitudes would require implausibly large surprisal values that are well outside the range of surprisal values we observe anywhere in our materials. In our paradigm, LM-predicted effects are approximately equal to the product of the difference in surprisal across conditions and the surprisal-to-reading-time regression coefficient estimated by the filler model. Assuming an optimistic surprisal-to-reading-time coefficient for Regressive Go-Past of 12.4 ms per bit of surprisal (the upper 97.5% percentile of the largest coefficient estimate in *SI Appendix*, Fig. S1, obtained from the RNNG), we would need to observe a surprisal difference of approximately 34 bits between the experimental and control condition in the classic garden path subset to predict even the lower bound of the empirical effect at the spillover word (which is 419 ms). But such a difference between the two conditions would require enormous surprisal values that are outside the range observed for our materials. The largest absolute surprisal value (as opposed to a difference across conditions) that we observe at any word in any of the experimental materials from the LM with the largest coefficient was 25 bits (for the distribution of LM surprisal values across all words, see *SI Appendix*, section 8). This means that even in the very unlikely case where the critical word were perfectly predictable in the unambiguous condition (i.e., its surprisal was zero), and were assigned the largest observed surprisal in the ambiguous condition, we still would only predict an effect of 25 bits × 12.4 ms/bit = 310 ms, which is substantially smaller than the lower bound of the empirical effect estimate.

Could other ways to extract surprisal from LMs close the gap between LMs and humans? In a recent study, Kuribayashi and colleagues (71) propose to derive surprisal from the internal layers of a transformer by attaching the language modeling head directly to the internal layer, complementing the standard method of deriving surprisal from the last layer (as we do). They show that surprisal derived from earlier layers better predicts early reading measures, whereas surprisal from late layers (including the last one) better predicts later measures. This suggests that surprisal derived in this way from early layers would provide an even better fit to Forward Reading Time than the last-layer surprisal we used, but it would most likely not close the gap we observe for the late measures; and surprisal from a later (but not last) layer would likely perform in a qualitatively similar way to the surprisal we derive from the last layer in our study.

## No Monotonic Function of Surprisal Can Simultaneously Explain Processing Difficulty in Syntactically Challenging and Simple Sentences

Following most prior work (23, 47), we have assumed that the relationship between surprisal and processing difficulty is linear. It is natural to ask if the gap between the human and LM-predicted effects would close if this relationship turned out instead to be superlinear, as proposed by ref. 72, for example. We next test if any monotonic relationship between surprisals and processing difficulty, such that higher surprisals are associated with increased difficulty, could predict the magnitude of the human effects. This simple, parameter-free analysis evaluates whether reading times and regression rates at the critical word in classic garden path sentences match those of words in the filler sentences with similar surprisal values. If reading times systematically diverge for filler words and critical words that are matched for their surprisal, we can conclude that no monotonic function of surprisal could explain reading times in both filler and garden path sentences simultaneously—any such a function would necessarily overpredict processing difficulty in filler sentences, or underpredict difficulty in garden path sentences.

We perform the analysis separately for the best LM in each family. We separate ambiguous garden path sentences into four equally sized bins based on the surprisal of the critical word. Then, we select all words from the filler sentences whose surprisal values fall into each of these bins, provided that they are in the same position in the sentence as any of the critical words in the garden path sentences, plus or minus one word. We then compute the mean reading time or regression probability of the words in each bin, and repeat this process for each measure. In Regressions Out and Regressive Go-Past Time, the effect of a word’s surprisal manifests more strongly at the following word than the current word, so we bin words based on the surprisal of the current word, but compute the means of these two measures at the following word, meaning we report mean reading measures at the spillover word for Regressions Out and Regressive Go-Past, and the critical word for the other two measures.

**Fig. 5.**
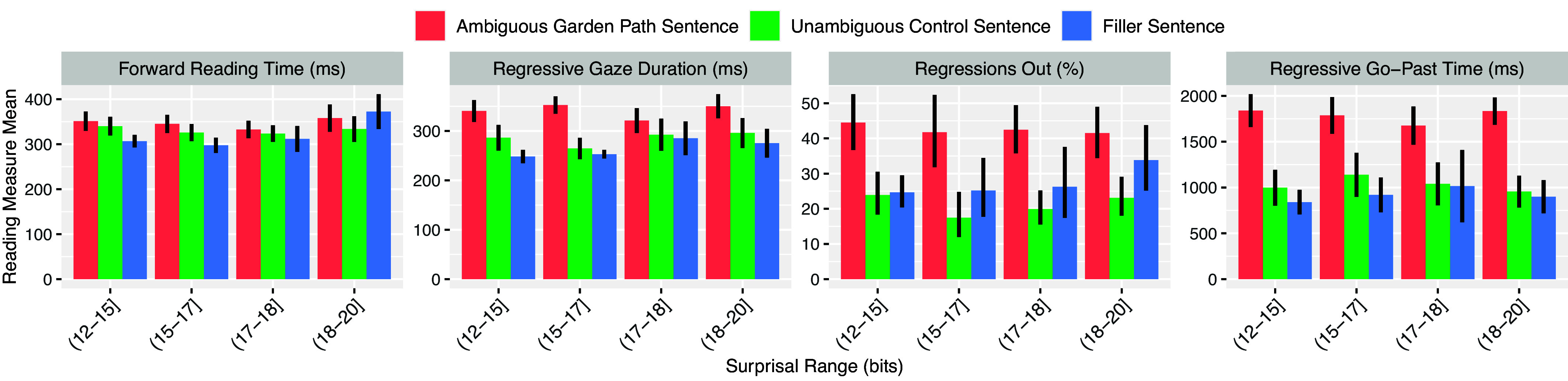
Reading times for a given surprisal value are consistently larger in garden path sentences than in naturalistic filler sentences, especially in regressive measures. Bars are reading measures for the critical word (Forward Reading Time and Regressive Gaze) or spillover word (Regressions Out and Regressive Go-Past) of ambiguous garden path sentences (red), binned by the surprisal assigned to that word by GPT-2, along with item-matched unambiguous control sentences (green), and surprisal-matched filler words (blue). Error bars represent 95% CIs adjusted for repeated measures. Methods for computing these CIs are described in *SI Appendix*, section 6.

We report results for GPT-2; the results for the other families are qualitatively similar (*SI Appendix*, section 6). Across the entire range of surprisal values, we find that filler words have much lower reading times and regression rates than surprisal-matched critical words of garden path sentences in the regressive measures (Fig. 5). For example, words with a surprisal between 12 and 15 bits have an average Regressive Go-Past duration of 802 ms [738–986 ms] in filler sentences, but an average Regressive Go-Past duration of 1,840 ms [1,600 to 2,012 ms] in the ambiguous conditions. This result suggests that the failure of LM surprisal to explain garden path effects is not due to the linear linking function assumed by our filler models: No monotonic linking function between LM surprisal and regressive reading measures can simultaneously capture the ambiguous garden path and filler data in Fig. 5. In *SI Appendix*, section 6, we show that this result cannot be attributed to differences in word length and frequency between the filler and garden path sentences.

## General Discussion

Prediction has been hypothesized to be central to cognition in general and language in particular. This hypothesis becomes even more alluring given the recent success of large language model-based AI systems, whose core language abilities emerge through a simple prediction-based objective—an objective that may well serve as one of the fundamental signals children use to learn the structure of language (e.g. refs. 73 and 74). While it is clear that word-by-word language processing difficulty in humans is closely correlated with word predictability—in particular with surprisal, the negative logarithm of the word’s probability—the precise cognitive work indexed by surprisal in language processing remains an active topic of investigation (23, 72, 75, 76). Understanding the limits of what can be explained by prediction is critical to the development of complete theories of language processing and cognition as a whole. In this work, we investigated whether a dissociation between prediction and other cognitive processes could be identified through the eye movements of readers in a large-scale benchmark dataset of reading behavior, containing both naturalistic and controlled experimental stimuli.

We conjecture that the sources of difficulty in naturalistic reading, which are well-explained by LM surprisal, are distinct from the sources of difficulty in temporarily ambiguous sentences, which are not. Consistent with this conjecture, we find a dissociation in the types of eye movements that LM surprisal does and does not capture when humans read syntactically challenging sentences. More specifically, LM surprisal can predict the full increase in a word’s reading time in contexts where it disambiguates the syntactic structure of a sentence, but only when fixations on the word are not followed by a regression (Forward Reading Time). This was based on surprisal estimates from over 400 LMs, with a wide variety of architectures and training data. This finding sheds light on earlier reports that surprisal underpredicts the magnitude of processing difficulty (28, 30, 35), and suggests that misalignments between LM surprisal and human processing difficulty likely do not reflect poor calibration between LM predictions and those of humans (29, 50). While such calibration issues always remain a possibility, the remarkable consistency of our findings across a diverse range of LMs, coupled with the good quantitative fit between LM surprisal and the effect of disambiguation on Forward Reading Time, suggests that the explanation for the misalignment likely lies elsewhere.

In our data, the misalignment between LM surprisal and human reading measures manifests in the rate at which critical words in these syntactically challenging sentences trigger rereading (Regressions Out), the reading time on a word preceding a regression (Regressive Gaze), and the amount of time spent rereading (Regressive Go-Past), but not in Forward Reading Time. The dissociation between different reading measures was particularly clear in classic garden path constructions, where the LM surprisal-based predictions of effect magnitudes in Forward Reading Time generally overlapped with the empirical effects but LM surprisal underpredicted effects in the rereading-based measures by orders of magnitude. In this way, our current study offers an important insight into the now well-established finding that surprisal underpredicts the cost of processing difficulty in syntactic disambiguation. Unlike self-paced reading and other behavioral reading methods used in prior work, the eye-tracking technique lets us separate trials into those with a regression, and those without. This proves critical. In the subset of trials where reading proceeds uninterrupted by a regression, LM surprisal can capture the processing difficulty associated with syntactic disambiguation. But when reading is interrupted by regressive eye movements and rereading, LM surprisal fails to capture the full magnitude of processing difficulty.

### Implications for Theories of Syntactic Disambiguation.

Sentence processing theories differ in whether they attribute syntactic disambiguation difficulty in garden path sentences to the costs of structural repair operations (i.e., reanalysis; 19, 77) or to the cost of probabilistic inference over a theoretically unbounded number of structures (4, 21, 78). The core difference between these two views is whether processing difficulty is driven by the cost of updating probabilistic beliefs over many possible meanings (what we refer to as prediction), or the cost of mentally assembling the meaning representations themselves (including structural reanalysis of the sentence after disambiguation). Our results are consistent with an account wherein both probabilistic inference and reanalysis contribute to the full cost of processing garden path sentences.

Our results dovetail with findings from neurolinguistic measurements that show a dissociation between prediction and structural processing (79–81). These studies have shown that surprisal and various indices of structure-building effort, defined relative to a parsing model, can capture distinct sources of variance in fMRI and EEG signals for naturalistic data. Our findings suggest that a dissociation between surprisal and structure-building (specifically, structural reanalysis) also obtains in reading time measures. The observation that LM surprisal can explain the full magnitude of garden path disambiguation in uninterrupted reading suggests that routine structure-building processes do not drive the extra processing difficulty above and beyond surprisal in our data. Instead, one implication of our results is that the severe processing cost in syntactic disambiguation is intermittent, arising only on the subset of trials with a regression; this in turn suggests that the shortfall between LM surprisal’s predictions and the observed processing difficulty is driven by a failure mode that arises on a subset of trials (82).

Our suggestion that different processes drive difficulty in trials with and without regressions parallels the results of an EEG and eye-tracking coregistration study (83), which found that neural responses characteristically differed on trials with regressive eye movements. In all trials, sentence-final anomalies elicited an N400 response, but trials with a regression also had a large P600 response, which has been linked to error detection (84, 85) or integration difficulty (86). Future work with coregistered eye movement and EEG recordings may help us better characterize which underlying comprehension processes LM surprisal can and cannot capture.

An open question for future research concerns what drives the integration failures that LM surprisal is unable to account for. One possibility is that garden path sentences lead comprehenders to create multiple, mutually incompatible interpretations of the stimulus over the course of comprehension, creating a representational conflict or competition that must be resolved (87). On this view, integration failures may result from the rapid detection of this conflict or incoherence, potentially cuing the comprehender to engage cognitive control processes to negotiate a determinate interpretation of the stimulus (for a recent review, see ref. 88). A related view, based in the cue-based parsing tradition, might be that integration failure is related to difficulty in the memory retrieval processes necessary to activate the relevant syntactic dependents in memory (for different models of this process, see refs. 37, 38, and 89). These models link intermittent parsing failures to an increased probability of a regression in reading. Another approach would be to invoke mechanisms such as limited beam search over parses (78) or particle filters (90), where some of the parses drop out of consideration if they are less likely. On this view, error detection, indexed by regressive eye movements, may stem from the recognition that there is no parse available that is compatible with the current input.

Any full model should aim to explain not only the probability of a regression, but also the amount of subsequent rereading following the regression (as reflected in Regressive Go-Past). This may reflect the work required to reconstruct a new parse after all of the parses had been eliminated through beam search, or the work needed to resolve the competition between conflicting interpretations. Future models of rereading should focus on how readers eventually update their syntactic (91, 92) or word representations (93, 94) as a result of rereading, and how that influences subsequent reading behavior. Our large-scale eye-tracking dataset will serve as a useful benchmark for the development of such future models of garden-pathing.

### Implications for Surprisal Theory.

How does surprisal relate to processing difficulty in reading? Our findings suggest two related but distinct possibilities. One is that surprisal captures the measurable costs of word recognition, but not any additional costs of integrating a recognized word with its context. For example, models of eye movements in reading such as E-Z reader (25) posit that word recognition is the primary determinant of forward reading behavior, and subsequent integration processes only affect eye movements when they fail (26). Under this account, modest slowdowns in Forward Reading Time in syntactically challenging and naturalistic sentences alike are driven by slowdowns in recognizing unexpected words, while the large slowdowns in the regressive measures reflect integration failures. This multistage account might be modeled by combining a probabilistic inferential model of word recognition like the Bayesian Reader (95, 96), with a separate model of structural integration costs. But a related possibility is that Forward Reading Time additionally reflects integration processes, but only when they are successful. On this view, surprisal indexes the cost of these successful integrations while falling short of capturing the large costs of error-driven disruptions.

Still, there are some limitations to the conclusions we are able to draw from our eye-tracking dataset. While we found that LM surprisal may explain the general order of magnitude of forward reading time at the condition level—certainly a lot better than any of the regressive reading measures—its fit was still not perfect. Despite the large number of subjects, the uncertainty in the estimates of construction level effects remained large. The uncertainty in the estimate of item-specific effects was even greater, especially in earlier measures, making it difficult to evaluate whether LM surprisal can explain the variability of the results across items (which was possible with self-paced reading, 35). This may be a fundamental limitation of eye-tracking, as getting meaningfully more precise empirical estimates of effect size would likely require many times more subjects than we collected.

## Conclusion

Reading is characterized by a dynamic pattern of forward and backward eye movements. Syntactically challenging sentences in particular are associated with longer reading times and a greater rate of rereading. Understanding this pattern can shed light on the cognitive processes driving language comprehension difficulty. We test two hypotheses as to what drives the eye movement pattern observed in syntactically challenging sentences. The first hypothesis explains it as a consequence of word prediction; the other argues that it is driven by two separate sets of processes, one involving surprisal, and another, later stage in which prior structural commitments are revised when errors are detected. Neural language models make it possible to rigorously test the extent to which these eye movement patterns can be fully explained by word surprisal. In a large-scale study that tracked eye movements from 368 human participants as they were reading syntactically challenging sentences, and that used surprisal estimates from hundreds of language models with different architectures and training setups, we find that estimates of a word’s contextual probability can capture forward reading behavior, in line with theories that attribute processing difficulty to probabilistic inference. By contrast, we find that rereading behavior cannot be straightforwardly explained by surprisal. While LMs have enabled us to make substantial progress in modeling word surprisal, surprisal estimates from those LMs cannot, on their own, explain human language comprehension; They need to be supplemented with equally accurate cognitive models of how words are integrated into a structured meaning when standard belief update processes fail.

## Materials and Methods

### Dataset Construction.

We collected eye-tracking data from human subjects that read items in four subsets (13 conditions in total)—the classic garden path subset, the relative clause subset, the attachment ambiguity subset, and the agreement violation subset—along with filler items. We describe all types of sentences in the remainder of this section. Each of the four subsets had 24 items, except for those in the agreement violation subset, which had 18 items. All items are identical to those used in ref. 35; a detailed list of the items can be found in *SI Appendix*, section 10.

#### Stimuli subsets.

##### Classic garden path subset.

The classic garden path subset contains three constructions, the Main Verb/Reduced Relative (MV/RR), Noun Phrase/Sentential Complement (NP/S), and Transitive/Intransitive (NP/Z) constructions. The ambiguous condition of each construction has a temporary syntactic ambiguity that is resolved at the critical word (in bracket). The corresponding unambiguous condition includes additional material that removes the syntactic ambiguity from the ambiguous condition.

1. **MV/RR Ambiguous**: The suspect sent the file [deserves] further investigation after the murder trial.
2. **MV/RR Unambiguous**: The suspect who was sent the file [deserves] further investigation after the murder trial.
3. **NP/Z Ambiguous**: Because the suspect changed the file [deserves] further investigation after the murder trial.
4. **NP/Z Unambiguous**: Because the suspect changed, the file [deserves] further investigation after the murder trial.
5. **NP/S Ambiguous**: The suspect showed the file [deserves] further investigation after the murder trial.
6. **NP/S Unambiguous**: The suspect showed that the file [deserves] further investigation after the murder trial.

In the MV/RR construction, when the verb *sent* is first read it is ambiguous between being the main verb of the sentence or an embedded verb in a relative clause modifying *the suspect*. When readers reach the actual main verb of the sentence, *deserved*, the sentence is disambiguated toward the relative clause interpretation. The addition of *who was* in the control condition makes it clear that the verb *sent* is unambiguously part of a relative clause.

In the NP/Z construction, the verb *changed* can either take a noun phrase complement (e.g., changed something) or no complement (intransitive: e.g., changed their clothes). Without the comma after *changed*, the following noun phrase *the file* is ambiguous between being a noun phrase complement of *changed* or the subject of another clause. The relativized verb *deserved* disambiguates the sentence in favor of the intransitive interpretation.

In the NP/S construction, the verb *showed* can either take a noun phrase complement (e.g., show something) or a sentential complement (e.g., show that something happened). Without the complementizer *that*, the complement *the file* is initially ambiguous between being a noun phrase complement, or the subject of a sentential complement. The second verb *deserved* disambiguates the sentence in favor of the sentential complement interpretation.

##### Relative clause subset.

The relative clause subset contains sentence pairs with lexically matched object-extracted relative clauses (ORCs) and subject-extracted relative clauses (SRCs).

7. **ORC**: The bus driver who [the kids followed] wondered about the location of a hotel.
8. **SRC**: The bus driver who [followed the kids] wondered about the location of a hotel.

Both ORCs and SRCs consist of a determiner (*the*), a noun (*kids*), and a verb (*followed*), which are the three word regions of interest. ORCs are more difficult to process than SRCs in many languages (97).

##### Attachment ambiguity subset.

The attachment ambiguity subset contains sentences with two noun phrases and a relative clause. The relative clause can modify the nearby noun phrase (low attachment), the more distant noun phrase (high attachment), or either noun phrase (ambiguous), depending on the number of each noun in the noun phrase.

9. **Low Attachment**: In the lobby, Clyde bumped into the chauffeurs of the CEO who [is] reckless and very unpopular with the company.
10. **High Attachment**: In the lobby, Clyde bumped into the chauffeur of the CEOs who [is] reckless and very unpopular with the company.
11. **Ambiguous**: In the lobby, Clyde bumped into the chauffeur of the CEO who [is] reckless and very unpopular with the company.

In the ambiguous condition, the critical word *is* can agree with either noun of the preceding noun phrase (here, either *chauffeur* or *CEO*). Previous studies report faster processing relative clauses where attachment is ambiguous compared to either low attachment or high attachment relative clauses (9, 98). Our items are modified versions of the stimuli used in ref. 99.

##### Agreement violation subset.

The agreement violation subset contains sentences where the verb does not agree in number with the subject:

12. **Violation**: If the supervisor changes, the schedules [deserves] further inspection by the rest of the staff.
13. **No Violation**: If the supervisor changes, the schedules [deserves] further inspection by the rest of the staff.

Unlike the sentences in the previous subsets, sentences containing an agreement error are simply ungrammatical, and it is not possible to derive a well-formed syntactic analysis from them, for example, by reanalyzing the critical word *deserves*.

##### Filler items.

The filler items are designed to elicit “typical” reading in the absence of processing difficulty due to syntactically challenging constructions. To this end, we selected 40 sentences from the Provo corpus (55), which contains text from news articles, magazines, and works of fiction.

#### Data collection and processing.

A total of 424 native speakers of English, with normal or corrected to normal vision, participated in the experiment. Participants were compensated with a $15 prepaid Visa gift card. The median duration of the experiment (measured as the total time the eye-tracker was recording, excluding setup and debriefing) was 36.4 min, with a lower 25th percentile of 31.5 min and an upper 75th percentile of 46.1 min. Pre-registered exclusion criteria include accuracy on the comprehension questions for the filler items (we excluded participants with accuracy lower than 80%, N=21) and excessive blinking rate on the target region of the critical trials (>25%, N=35), leaving 368 participants’ data in the analysis.

Data were collected across four universities (UMass Amherst, NYU, JHU, and Colgate), using the same equipment and experimental setup. The experimental protocol was approved by each university’s institutional review board (UMass Amherst: #3736; NYU: #FY2020-4512; JHU: #HIRB0005932; and Colgate: #FR-F22-05). Informed consent was obtained from all participants, who read and signed a form prior to participation outlining the study’s purpose, procedures, risks, benefits, rights to withdraw, confidentiality, compensation, and points of contact. Participants were seated in a quiet room with a 20 inch LCD monitor positioned 60 cm in front of them. Each sentence was presented on a single line in 12-point monospaced font (1^°^ visual angle fitting about 3 to 4 characters). Participants were instructed to read each sentence naturally and answer a question that followed, while maintaining their head on the SR Eyelink 1000 tower-mount eye-tracker. The movement of the right eye was recorded at a sampling rate of 1,000 Hz. Three-horizontal-dot calibration was performed at the beginning of the experiment and reconducted between trials if the average drift from the previous calibration exceeded 0.3, if the maximum drift exceeded 0.5, or if the participant noticeably moved their during/between trials.

Following ref. 35, the four subsets were distributed into 18 experimental lists using a Latin square design. Each participant only read four unique items in each of the 13 experimental conditions (52 trials). Each list was combined with the same 40 filler items in a single experimental session. The items in the agreement subset always occurred in the last 18 trials of the session (embedded with 10 filler items); this was done to make the reading of the remaining items as natural as possible, since these items were the only ones that contained grammatical errors. No items from two paired conditions (e.g., NP/S ambiguous and NP/S unambiguous) were presented consecutively.

Data were processed using the UMass eye-tracking Lab software.^*^ A fixation whose duration was shorter than 80 ms and within 1 character from the preceding or following fixation was combined with that fixation. We excluded from analysis trials with gaze duration on the critical word of 2,000 ms or more.

## Supplementary Material

Appendix 01 (PDF)

## Data Availability

All data and code used in our analyses are available at https://osf.io/b6rqh/ (100). All other data are included in the manuscript and/or *SI Appendix*.

## Footnotes

^*^Eyetrack version 7.10m: https://websites.umass.edu/eyelab/software.
