# Supplementary material for: Eye movements reveal a dissociation between prediction and structural processing difficulty in language comprehension
Source: Proc Natl Acad Sci U S A. 2026 Aug 7;123(32):e2532230123. doi: 10.1073/pnas.2532230123 (PMC13462876; doi:10.1073/pnas.2532230123)
Supplement: Supplementary file 1 [file pnas.2532230123.sapp.pdf]

PNAS

Supporting Information for

Eye movements reveal a dissociation between prediction and structural processing difficulty in language comprehension

William Timkey, Kuan-Jung Huang, Byung-Doh Oh, Grusha Prasad, Suhas Arehalli, Tal Linzen, Brian Dillon

William Timkey
E-mail: wpt2011@nyu.edu

This PDF file includes:

Supporting text
Figs. S1 to S18
SI References

Contents

1 Overview of the evaluated LMs **3**

2 Why we use regression-contingent eye-tracking measures **3**
A Results from non-regression-contingent measures 4
A.1 Gaze duration 4
A.2 Go-past time 4

3 Analysis of filler models **5**
A Filler model surprisal coefficients 5
B Filler model goodness-of-fit 5

4 Results from other constructions **5**
A Subject-verb agreement violation 5
B Attachment ambiguity 8

5 Bayes Factor analysis of LM-human alignment **8**

6 Detailed model results for "*No monotonic function of surprisal can simultaneously explain processing difficulty in syntactically challenging and simple sentences*" **11**

7 Detailed methods for "*LM surprisal explains forward reading but not rereading*" **11**
A Estimating effects due to syntactic disambiguation 11
A.1 Empirical effects from human data 11
A.2 Predicted effects from LM surprisal 12
B Regression model formulae 13

8 Distributions of LM surprisal values **17**

9 Influence of LM size and amount of training data on garden path effect magnitudes **17**

10 Full list of experimental sentences **28**
A Classic garden path subset 28
B Relative clause subset 30
C Attachment ambiguity subset 31
D Agreement violation subset 33
E Filler items 34

Supporting Information Text

1. Overview of the evaluated LMs

We generated predictions of reading measures from a total of 409 LMs from six model families that span different architectures, training objectives, parameter counts, and training data compositions and scales. Each sentence was presented to each LM in isolation, without any preceding or following context. The `Pythia`, `Mamba`, `GPT-2` and `lossy-context` models use subword tokenization, and the other models use whitespace tokenization. We compute a word's surprisal as the sum of the surprisal of all of its subtokens. For subword tokenized models, we applied a correction to account for the probability of the trailing whitespace as part of each word's probability to ensure a consistent probability distribution (1, 2). Where we report just a single measure per model family in the main paper, we show the effects predicted by the LM from each of the six model families that achieved the best fit to the *filler* reading data across the four reading measures (that is, models were not selected based on their fit to the experimental sentences). The goodness-of-fit to the filler reading data was measured as the increase in likelihood due to including LM surprisal over a regression model that contains only the baseline predictors.

The model families we evaluated are:

Pythia (3): A family of transformer-based (4) autoregressive language models trained on the next-word prediction objective over ∼300B tokens from the Pile dataset (5). The Pythia family spans a wide range of model parameters (eight sizes with ∼70M, ∼160M, ∼410M, ∼1B, ∼1.4B, ∼2.8B, ∼6.9B, ∼12B parameters), with checkpoints of each model available at various stages of training, when they have seen different amounts of training data. We evaluated models of all eight sizes at 19 stages during training (when models have seen 1, 2, 4, 8, 16, 32, 64, 128, 256, 512, 1000, 2000, 4000, 8000, 16000, 32000, 64000, 128000, 143000 training batches of ∼2M tokens), resulting in a total of 152 models.

GPT-2 (6): A family of transformer-based autoregressive language models trained on the next-word prediction objective over 40GB of internet text. We evaluated the four officially released GPT-2 models, which vary in their number of parameters (∼124M, ∼355M, ∼774M, ∼1.6B parameters).

Mamba (7): A family of autoregressive language models based on the Mamba architecture (7), which rely on recurrence-like operations that are constrained to be parallelizable. These models were trained on the next-word prediction objective over ∼300B tokens from the Pile dataset (5). We evaluated models of all five sizes released by the authors, with ∼130M, ∼370M, ∼790M, ∼1.4B, and ∼2.8B parameters.

LSTM (vS) (8): A family of language models based on the long short-term memory architecture (LSTM; 9), trained on the next-word prediction objective over the WikiText-103 dataset (10). We evaluated all 125 released models; these included all combinations of five different sizes (with 100, 200, 400, 800, 1600 hidden units in each of the two LSTM layers), five different amounts of training data (2M, 10M, 20M, 40M, 80M words), and five distinct subsets of the WikiText-103 dataset.

RNNG: A family of recurrent neural network grammar (RNNG; 11, 12) models. These are recurrent neural networks that explicitly build syntactic structure by jointly predicting parsing operations and the upcoming word (unlike standard recurrent neural networks, which only predict the upcoming word). These models require a parsed version of the training corpus. We trained 10 models on a 42M-word machine-parsed subset of the BLLIP corpus (13), using all combinations of five different random seeds and two parsing methods (top-down parsing and left-corner parsing). We then calculated surprisal estimates from these 10 models using word-synchronous beam search (14) with 12 different beam sizes (1, 2, 3, 4, 5, 10, 25, 50, 100, 250, 500, 1000 states), resulting in a total of 120 models.

LSTM (G) (15): A two-layer LSTM language model with 650 hidden units on each layer, trained on the next-word prediction objective over 90M tokens from the English Wikipedia.

Lossy-context (16, 17): A resource-rational lossy-context surprisal model implemented by (17). This model implements the lossy-context variant of surprisal theory (18), designed to model effects of limited working memory on prediction. The model assumes that tokens in context are erased at an overall rate of p. The model learns to optimally retain the tokens that are most useful for predicting the next word. The model then attempts to reconstruct the original context from this lossy representation, and predict the next word. Concretely, we use `GPT-2 medium` as the "base model" which handles the next-word prediction, and we train three random seeds of the noisy memory model at two deletion rates: 20% and 50%. Inference is performed over four iterations within each seed, as recommended by (17); the twelve surprisal estimates at each word (four per seed) are averaged into a single surprisal value per word. The model uses the standard `GPT-2` subword tokenization scheme.

2. Why we use regression-contingent eye-tracking measures

The eye-tracking measures we use are Forward Reading Time, Regressive Gaze Duration, and Regressive Go-Past Time, which are regression-contingent (19–22) versions of the common measures Gaze Duration and Go-Past Time (23). The "classic" (non-regression-contingent) measure Gaze Duration is the sum of all first-pass fixations on w_i before it is exited in either direction, and is equivalent to Forward Reading Time when w_i is exited to the right, and Regressive Gaze Duration when exited to the left. Likewise, the non-regression-contingent measure Go-Past Time is the sum of all fixations on any word from the first fixation on w_i until any word $w_{>i}$ is fixated, and is equivalent to Forward Reading Time when w_i is exited to the right, and Regressive Go-Past Time when exited to the left.

We chose to analyze the regression-contingent versions of these more common measures for several reasons. Empirically, Gaze Duration is known to be significantly longer when words are exited via a forward eye movement than when they are exited via a regression (20–22). One intuition for this pattern is that error-driven saccades will occur as soon as a processing error is detected, possibly before w_i is fully processed, while forward saccades from w_i occur after w_i has been fully processed.

In addition, models of eye-movement control during reading such as E-Z Reader 10 (24) argue that forward saccades are often triggered when only the first of two lexical processing stages is complete, and that processing errors and regressive saccades due to integration failure can manifest on the following word (w_{i+1}). Processing difficulty is known to manifest as increases in REGRESSIONS OUT and as increases in GAZE DURATION (24–26), but it is also the case empirically that increases in REGRESSIONS OUT are associated with *decreases* in GAZE DURATION (19–22). Thus, differences in the rate of regressive saccades across conditions can mask an underlying positive effect in GAZE DURATION (19), or create a negative effect when none is in fact present, if GAZE DURATION is aggregated across both regressive and non-regressive trials. To avoid this confound, we separate this measure into two—FORWARD READING TIME and REGRESSIVE GAZE DURATION—based on whether or not a regression has occurred.

The non-regression-contingent GO-PAST TIME measure likewise confounds the rate of regressions with the duration of rereading. If we were to observe an increase in GO-PAST TIME, it would not be clear whether this effect is simply driven by an increase in the rate of regressions, or whether it also reflects an increase in the duration of rereading given that readers have regressed. Because our goal is to assess the ability of LM surprisal to differentially explain distinct reading measures—separating the rate of regression from rereading time given that a regression has occurred—we analyze GAZE DURATION and GO-PAST TIME in a regression-contingent manner, which eliminates the confound with regression rate.

A. Results from non-regression-contingent measures. We next present results from an analysis of the non-regression-contingent aggregated measures (GAZE DURATION and GO-PAST TIME). Results from all constructions and all measures, including the non-regression-contingent measures, are summarized in Figure S3.

A.1. Gaze duration. In the classic garden path constructions, empirical effects in GAZE DURATION appear to reflect a mixture of the patterns in REGRESSIVE GAZE DURATION and FORWARD READING TIME. In all classic garden path constructions, the effect is localized to the critical word (MV/RR: 30 ms [17 to 44 ms], NP/Z: 24 ms [9 to 40 ms], NP/S: 17 ms [3 to 31 ms]). The predicted effects of several LM families overlapped with the empirical effects in the NP/Z and NP/S constructions, the closest of which was `GPT-2`, with a predicted effect of 14 ms [8 to 19 ms] in the NP/Z construction and 7 ms [2 to 12 ms] in the NP/S construction. All LMs underpredicted the magnitude of the empirical ambiguity effect in the MV/RR construction, with the largest LM-predicted effect being 9 ms [6 to 13 ms] from `GPT-2`. There was no detectable difference in the magnitude of the empirical ambiguity effect across constructions (MV/RR vs. NP/Z: [-25 to 13 ms], MV/RR vs. NP/S: [-32 to 5 ms]).

In the relative clause subset, there was no detectable empirical effect at the determiner region [-2 to 22 ms], but there was a negative effect (i.e., a subject relative clause advantage) at the noun region of -39 ms -[51 to 27 ms]. The LM-predicted effect of several models at this region was in the opposite direction of the empirical effect. There was also an empirical effect at the verb region of 17 ms [5 to 30 ms], which overlapped with the predictions of several LMs, though the LM-predicted effect appears to be driven by baseline predictors rather than LM surprisal, as the LM surprisal models' predictions did not differ substantially from those of the baseline model. The negative empirical effect at the noun region is particularly puzzling given that an empirical effect in the opposite direction at this region is observed in both REGRESSIONS OUT and GO-PAST TIME. An identical pattern was observed by (27), which used identical materials to those in the present study. Staub suggests that this pattern may be driven by the increased rate of regressions at the noun region, as GAZE DURATION tends to be shorter when fixations on a word are followed by a regression compared to a forward saccade. Indeed, when we control for regressions as in our regression-contingent analyses, we show that the magnitude of the effect at the noun in both FORWARD READING TIME and REGRESSIVE GAZE DURATION is substantially reduced. The remaining effect in the regression-contingent analyses may still be driven by an increase in regressions at the PREVIOUS word. The regression-contingent analyses we report in the main body of the paper ensure that any effect driven by rates of regressions manifests only in REGRESSIONS OUT, and does not confound the other measures.

A.2. Go-past time. At the critical region of the classic garden path subset, GO-PAST durations were 262 ms [196 to 325 ms] higher in the ambiguous conditions (757 ms [684 to 830 ms]) than the unambiguous conditions (467 ms [436 to 499 ms]) when averaged across the three constructions. As with REGRESSIVE GO-PAST TIME, all LMs drastically underpredict the magnitude of this effect. The largest LM-predicted effect was 24 ms [18 to 30 ms] from the `Pythia` model. The ambiguity effect was significantly larger in the MV/RR construction (276 ms [209 to 340 ms]) than in the NP/S construction (177 ms [88 to 266 ms]), but there was no evidence for a difference between the ambiguity effect in the MV/RR and NP/Z (332 ms [228 to 436 ms]) constructions. Among the LMs, only the `RNNG` model predicts this difference across constructions. At the first spillover word of the classic garden path subset, there was a large ambiguity effect of 415 ms [308 to 514 ms] across all constructions. This effect was significantly larger in the MV/RR construction (544 ms [436 to 644 ms]) and the NP/Z construction (523 ms [380 to 644 ms]) than the NP/S construction (178 ms [50 to 303 ms]). The LMs once again drastically underpredict the magnitude of the ambiguity effects at the spillover word. The largest predicted ambiguity effect at the spillover word was 33 ms [24 to 41 ms] in `GPT-2`, twelve times smaller than the empirical effect. In the classic garden path constructions, the empirical pattern of results in GO-PAST TIME was largely similar to that observed in REGRESSIVE GO-PAST TIME, suggesting that the empirical effects in GO-PAST TIME are not just driven by increased *frequency* of regressions in the ambiguous condition, but also increased *duration* of rereading given that a regression has occurred.

In the relative clause subset, there was a positive empirical effect (i.e., a subject relative clause advantage) at the determiner region of 124 ms [88 to 159 ms]. The largest LM-predicted effect was only 20 ms [4 to 36 ms] from the `Pythia` model. There was also an empirical effect in the same direction at the noun region of 191 ms [153 to 228 ms]. The effect at this region

was also underpredicted by LM surprisal. The largest LM-predicted effect at this region was 41 ms [33 to 49 ms] from `GPT-2`. The effect at the verb region trended in the negative direction, but its 95% credible intervals overlapped with zero. The LM-predicted effects at the verb region were all positive, but this appears to be driven by the baseline predictors, as the baseline model predictions did not differ substantially from the predictions of the LM surprisal models.

3. Analysis of filler models

A. Filler model surprisal coefficients. The current word surprisal coefficients in the best filler model from each LM family are presented in Figure S1. We report both *raw* surprisal coefficients (i.e., the increase in milliseconds of reading, or increase in the log-odds of a regression, associated with a one bit increase in LM surprisal) and *scaled* surprisal coefficients (i.e., the number of standard deviations increase in the reading measure associated with one standard deviation increase in LM surprisal). Scaled coefficients generally quantify the strength of association between LM surprisal and the reading measure. We present scaled coefficients to allow for direct comparison across LMs and measures. We find that scaled surprisal coefficients are consistently larger in Forward Reading Time than in Regressive Gaze or Regressive Go-Past, suggesting that LM surprisal is more strongly associated with Forward Reading Time than the other measures.

B. Filler model goodness-of-fit.

In filler sentences, LM surprisal explains earlier measures better than later ones. Before testing LM predictions for the targeted experimental stimuli, we first evaluated the goodness-of-fit of the filler models for each LM on each reading measure. We quantify goodness-of-fit as the increase in the log-likelihood (ΔLL) of the filler reading measurements when LM surprisal is included as a predictor in the regression model, compared to a regression model for the fillers with only baseline predictors, without LM surprisal (28, 29).

In the models fit to the filler sentences, LM surprisal is a significant predictor of all four measures in at least one model of each of the six LM families we investigated. How much variance is explained by LM surprisal? We consider this both in absolute terms, comparing absolute ΔLL across the four eye movement measures, and relative to the ceiling of explainable variance in each eye movement measure. Because we have a different number of observations for each measure (since they are conditioned on the direction of the saccade following the word), we divide ΔLL by the number of observations we have before comparing it across measures.

A standard measure of the goodness-of-fit for a regression model is the log-likelihood the response data given the model. To evaluate how much a single predictor improves a model's fit to the data, we can take the difference in the log-likelihood (ΔLL) of a regression model with only baseline predictors, and a model that also includes the predictor of interest (30). In this case, the response data is reading data from a particular eye-tracking measure, and the predictor of interest is surprisal from a particular LM. Concretely, the goodness-of-fit of surprisal in a given LM and reading measure is the difference in log-likelihood (ΔLL) of the filler reading data in a full regression model with both LM surprisal and baseline predictors, and a regression model with only baseline predictors.

To contextualize the goodness-of-fit of LM surprisal, we also compute a theoretical upper-bound ΔLL for each reading measure, which takes into account the intrinsic noise of the measure, and the fact that a single word-level difficulty estimate like surprisal cannot account for participant and trial-level variance. To compute this upper-bound, we follow methods for computing variance ceilings from prior work (31, 32). First, we randomly split our 368 participants into two equally sized subsets, A and B. For each measure, we compute the per-word mean reading time or regression rate across all participants in subset A, and then use this as a predictor instead of surprisal in a new filler model fit to reading data from all participants in subset B; see Section B for the upper-bound filler model formula. We then compute the ΔLL of the subset B data between a full model (i.e., a model with the per-word mean from subset A as a predictor), and a baseline filler model (with an identical formula to all other baseline filler models) fit to the same data. Next, we repeat this process by computing word-level mean reading times from subset B and fitting the filler model to the subset A data. Finally we sum the ΔLL from both subsets to get the total ΔLL. This process is repeated for 15 iterations with different random participant splits. We report the per-observation ΔLL, averaged across these 15 iterations.

The results are summarized graphically in Figure S2. Among the four reading measures, LM surprisal provides a better fit to Forward Reading Time than any other measure (Figure S2), in both absolute and relative terms (0.004 and 25% respectively), though in relative terms it is closely followed by Regressive Gaze Duration (absolute: 0.0016; relative: 24%). In absolute terms, LM surprisal attains the second largest ΔLL in Regressive Go-Past Time, but there is a great deal of explainable variance in this measure that was not captured by LM surprisal, with the best LM attaining only 9% of the variance ceiling. Absolute ΔLL is lowest in Regressive Gaze Duration, but there is high variability in this measure across participants, leading to a low variance ceiling.

Overall, in the filler sentences LM surprisal is a stronger predictor of the earlier reading measures Forward Reading Time and Regressive Gaze Duration than the other measures. In both cases it leaves most of the variance unexplained, but the proportion of unexplained variance is considerably larger in the later measures.

4. Results from other constructions

A. Subject-verb agreement violation. We find no evidence in this subset for any effects of agreement violations in Gaze Duration, or in its regression-contingent counterparts of Forward Reading Time and Regressive Gaze Duration. We

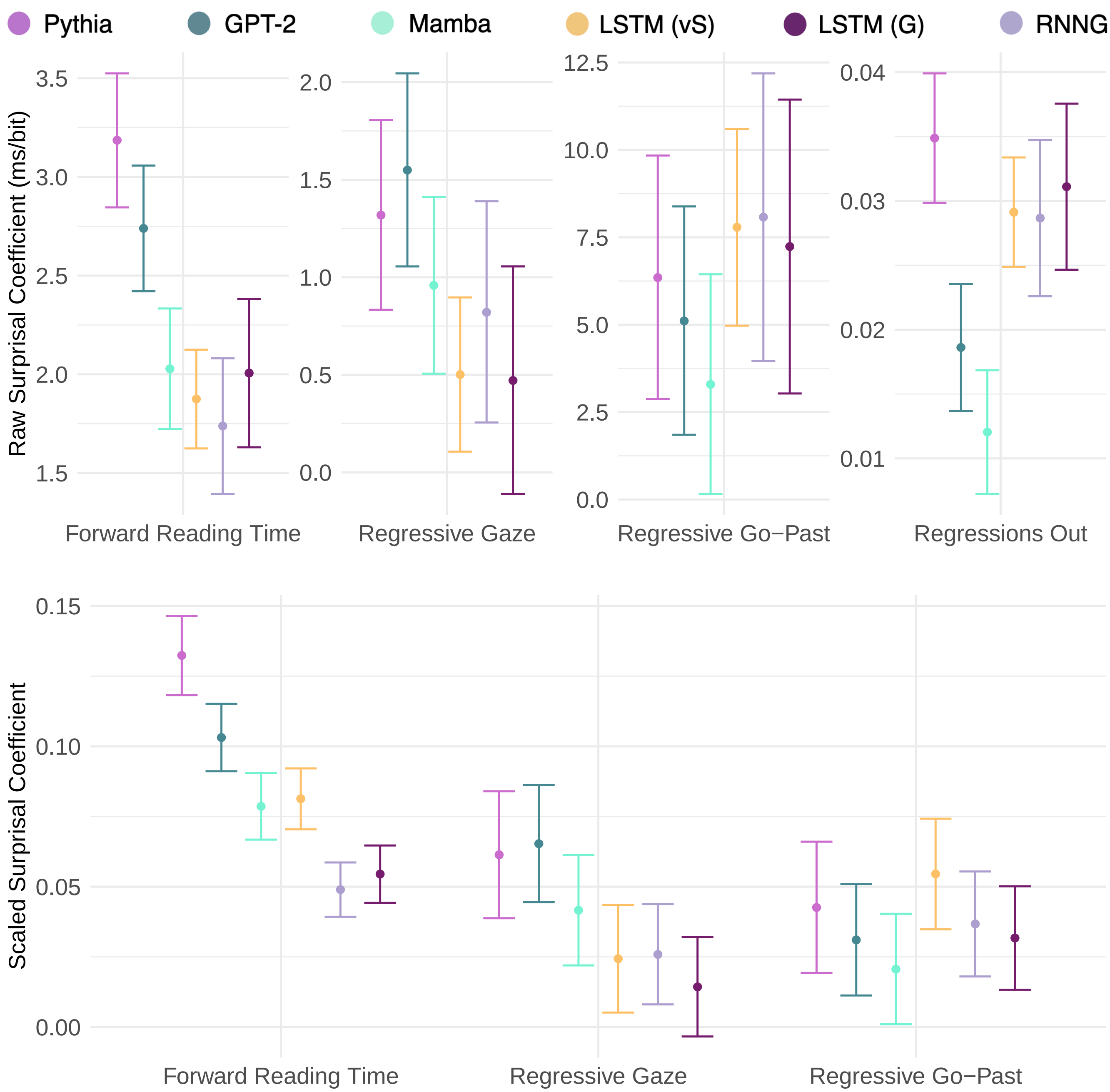

Fig. S1. Current word raw surprisal coefficients (top) and scaled surprisal coefficients (bottom) from filler models of six representative LMs, grouped by reading measure. Each point in this figure represents a coefficient from a single filler model, which is trained to predict one of the four reading measures using surprisal values from a single LM. The raw coefficients quantify the expected increase in milliseconds of reading time (or log-odds of a regression in the case of REGRESSIONS OUT) of a word as that word's surprisal increases by one bit. The scaled coefficients quantify the expected increase in standard deviations of reading times as surprisal increases by one standard deviation. Error bars represent bootstrapped 95% confidence intervals. Note that only scaled surprisal coefficients can be directly compared across LMs and measures.

do, however, find an effect in REGRESSIONS OUT. The rate at which readers regressed was 50% [24 to 79%] higher in the agreement violation condition than the no-violation condition. Interestingly, the predicted effect from `GPT-2` of 29% [26 to 31%] overlaps with the human results. This was the second largest effect in REGRESSIONS OUT predicted by `GPT-2` across all constructions (behind only the predicted ambiguity effect in the NP/Z construction), yet the magnitude of the empirical effect was much smaller than many of the effects seen in the other constructions. It is possible that the trigger for regressions in agreement violation configurations might be distinct from that of garden path configurations, and that the former might be better explained by word surprisal than the latter.

Results for GO-PAST TIME and REGRESSIVE GO-PAST TIME are more consistent with the general pattern observed in the other constructions, where LM surprisal cannot explain the full magnitude of the effect. In GO-PAST TIME, there was an

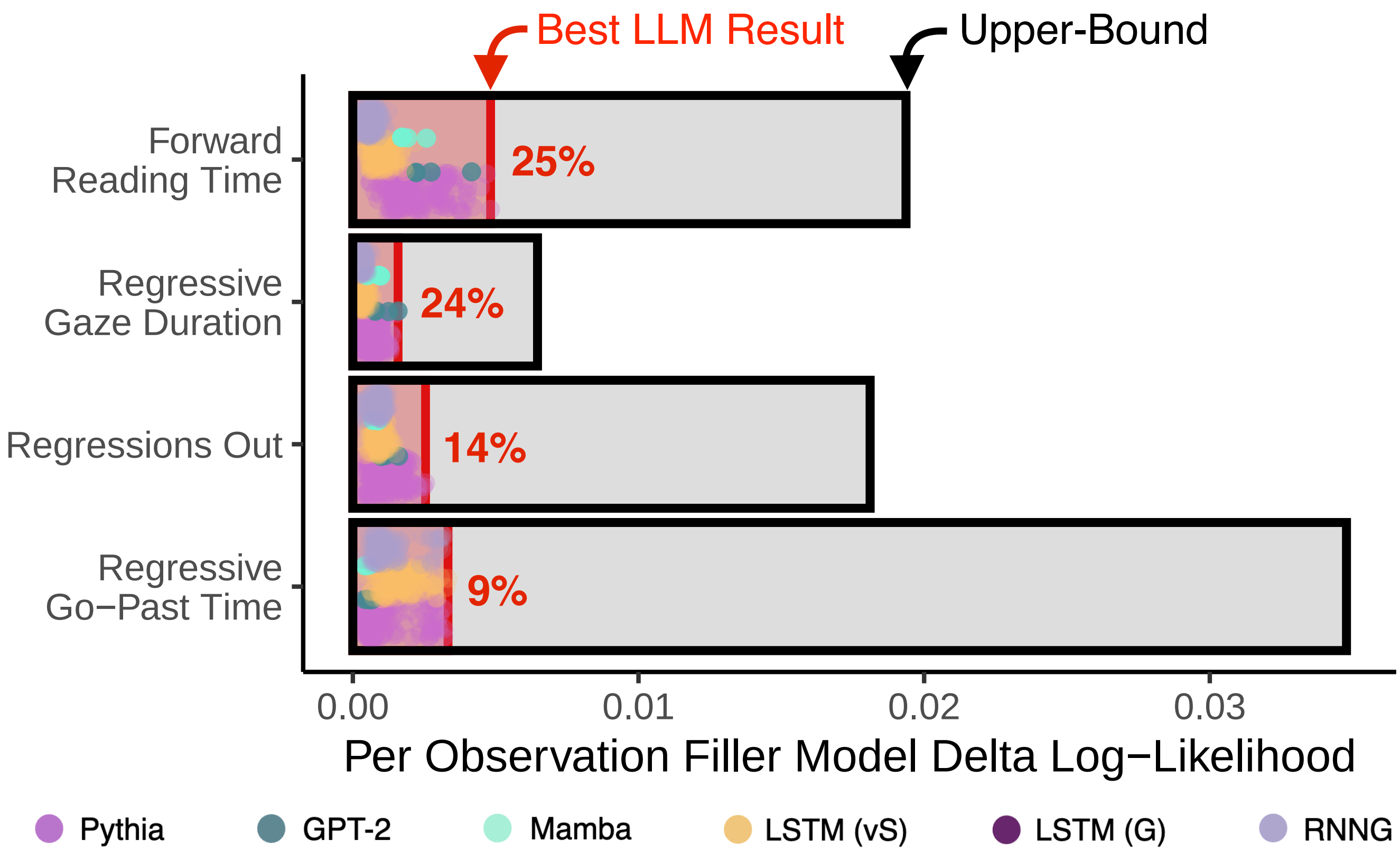

Fig. S2. Goodness-of-fit of LM surprisal in filler models for each of the four eye-tracking measures, measured as delta log-likelihood (△LL) of the filler data over the no-surprisal baseline filler model. Because filler models for different measures are fit to a different number of observations (depending on the direction of the saccade exiting the word), we divide the total △LL by the number of observations each filler model is fit to, where each observation is a measurement for a single word from a single trial. Each dot corresponds to one of the 409 LM-based surprisal estimates. A red line denotes the highest △LL attained by any LM for a given measure, and the upper bound on △LL is denoted by gray bars.

empirical effect of 168 ms [94 to 240 ms] at the critical word, and a smaller effect of 69 ms [8 to 130 ms] at the spillover word. The closest LM-predicted effect at the critical word was 46 ms [42 to 49 ms] from `GPT-2`. No LM predicted the existence of an effect at the spillover word. In Regressive Go-Past Time, there was an empirical effect at the critical word of 272 ms [102 to 433 ms], which was larger than that observed in the non-regression-contingent measure. Interestingly, only two LMs predicted any effect at the critical word, the larger of which was 26 ms [13 to 41 ms] from the `LSTM (vS)`. The LM predictions in Regressive Go-Past Time were not only well below the empirical effect magnitude, but were also smaller than the predicted effect in the non-regression-contingent Go-Past measure, opposite to the empirical pattern.

B. Attachment ambiguity. Evidence for ambiguity effects in the attachment ambiguity subset was limited. Empirically, we observe ambiguity effects only in Regressions Out and Go-Past Time, and only at the spillover word. These effects were relatively small in magnitude compared to effects in the other constructions. Regression rates were 23% [3 to 47%] higher in the unambiguous condition than the ambiguous condition, and Go-Past times were 80 ms [19 to 139 ms] longer. This was a similar pattern to the self-paced reading results reported in (32), where no ambiguity effect was found in the low attachment condition, and the ambiguity effect in the high attachment condition was smaller in magnitude than the effects observed in any other constructions. In Regressions Out, 95% credible intervals for the LM-predicted effects overlapped with those of human effects in all LMs. The largest predicted effect was 10% [8 to 13%] for `GPT-2`. In Go-Past Time, only the effect predicted by `GPT-2` (19 ms [14 to 24 ms]) overlapped with the empirical effect.

5. Bayes Factor analysis of LM-human alignment

To test whether the surprisal-predicted effects differ from empirical effects, we fit Bayesian regression models that quantify the remaining magnitude of the empirical effect not explained by surprisal. This quantity should be equal to zero if LM-predicted reading times capture the full magnitude of the experimental effect (the null hypothesis), and greater than zero if surprisal underpredicts the effect magnitude (the alternative hypothesis). Concretely, we quantify the relative evidence in favor of LM surprisal capturing an effect by fitting Bayesian regression models that predict a word's reading time from two predictors: the LM surprisal-predicted reading time, and a binary predictor indicating the experimental condition. Crucially, the coefficient of the LM-predicted reading time is fixed to one, making the coefficient of the condition predictor quantify the magnitude of the residual empirical effect that cannot be explained by the LM-predicted reading times. The resulting coefficient for the effect of interest (e.g., "`ambiguity`" in the case of the classic garden path subset) should be equal to zero if LM-predicted reading times capture the full magnitude of the experimental effect (the null hypothesis; $\beta = 0$), and not equal to zero if the empirical effect differs in magnitude from the LM-predicted effect (the alternative hypothesis; $\beta \neq 0$). Because the Bayesian regression models used to estimate the Bayes factors take a considerable amount of time to train, we conducted this analysis for only the single LM instance for each measure whose filler model attained the best fit to the filler data (quantified as ΔLL).

We quantify the relative evidence in favor of the null hypothesis vs. the alternative using Bayes factors (BFs; 33), which are the ratio of the likelihood of the reading data under the null hypothesis against the marginal likelihood under a range of plausible alternative effect sizes. We compute these Bayes factors as the inverse of the Savage-Dickey density ratio at $\beta = 0$. For the sake of readability in our plots and numerical stability issues for extremely large Bayes factors, for each Bayes factor b, we report $min(b, 100000)$. In our case, a BF of less than one indicates that evidence favors the null, and a BF of greater than one indicates that evidence favors the alternative. Because Bayes factors are highly sensitive to the specified priors, modelers often compute multiple BFs using different prior beliefs about the range of plausible effect sizes σ under the alternative hypothesis. If our prior belief is that differences between LM surprisal-predicted and empirical effects are underlyingly small, then we require very strong evidence of equality in order to accept the null. If our prior belief is that LM-predicted and empirical effects can diverge drastically, then we can accept the null with weaker evidence of equality.

In this case, we did not have strong priors for the range of plausible effect sizes, so we elected to take the approach outlined by (33). We evaluate a range of priors with varying degrees of vagueness (specified by a prior on the standard deviation σ on the distribution of effect sizes). Because each of our measures have different means and variances, a given σ value likely does not correspond to the same level of vagueness across all measures. Various works (33, 34) suggest in cases like this that the effect size σ prior be chosen as some fraction of the pooled sample standard deviation (similar to measures of standardized effect size like Cohen's d). In our case, a good candidate is the root mean squared error (RMSE) of the LM+measure's filler model, given that the residual standard deviation quantifies how far the filler model's predictions are on average from the actual observations in the filler data. We would not expect the difference in LM-predicted and empirical effect sizes to be larger than the average difference between LM-predictions and empirical RTs in a single observation. This represents the most vague alternative, where the effect size σ's prior is equal to 100% of the filler model RMSE. The strictest prior we evaluate is 5% of the filler model RMSE, which is likely too strict to be realistic, but nevertheless shows the behavior of the Bayes factor as the range on possible effect sizes goes to zero, as advocated by (33). For logistic regression models, we used the method established in (35) for computing standard effect sizes in logistic regression for the same effect sizes as in the reading time measures. Concretely, the prior on effect size σ for the vaguest alternatives were 114 ms for Forward Reading Time, 107 ms for Regressive Gaze, 716 ms for Regressive Go-Past, 115 ms for Gaze Duration, 420 ms for Go-Past and 1.81 for Regressions Out.

If the Bayes factor consistently favors the null (i.e., is less than one) as the vagueness of the alternative approaches zero, then the null is "unbeatable". It is important to not make strong conclusions based only on the BF for the strictest alternative, but to also evaluate the magnitude of the Bayes factor for more realistic priors on the effect size. According to (36), evidence

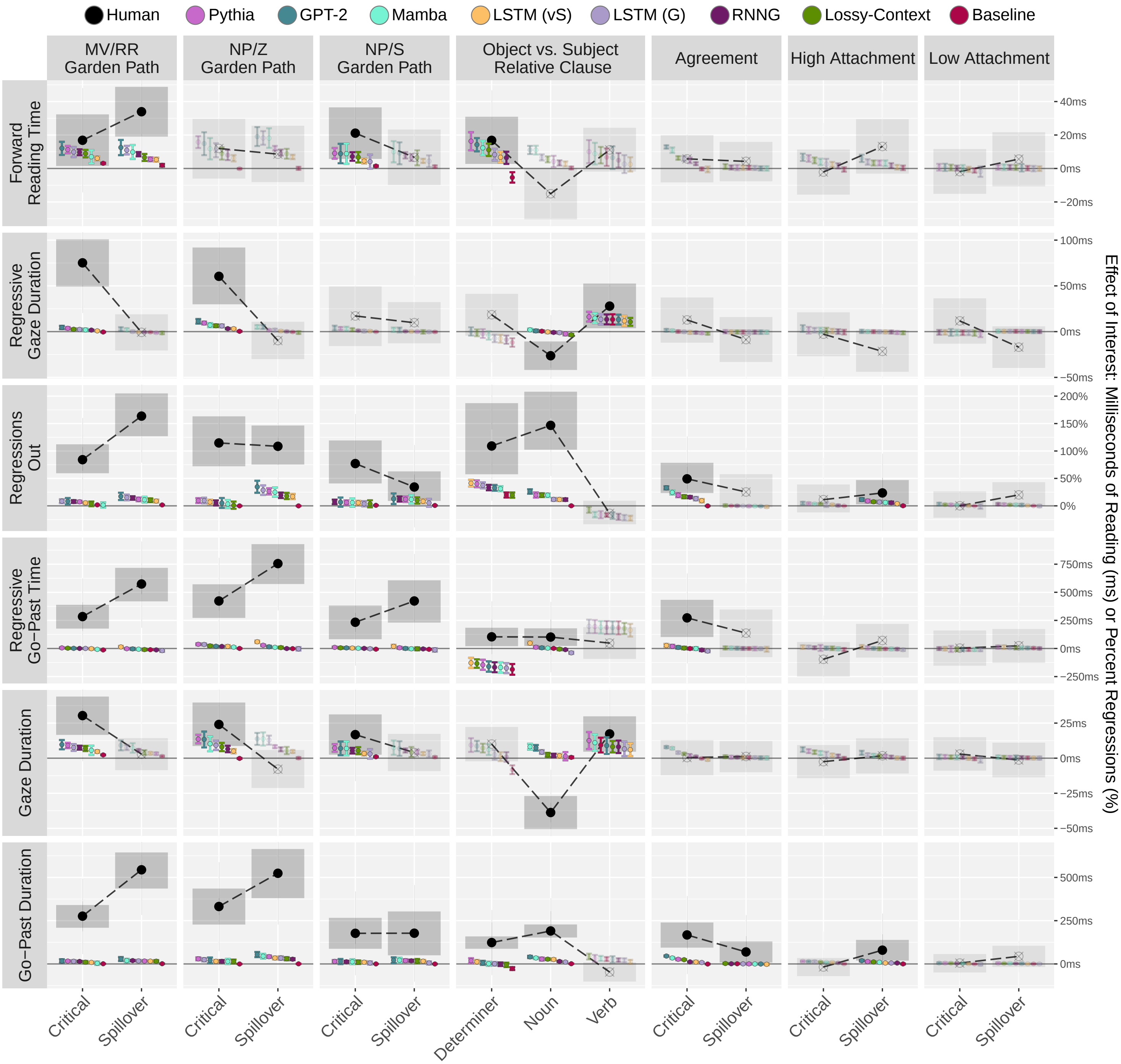

Fig. S3. Empirical (human) and predicted (LM) effects of interest at the regions of interest in all constructions (columns) and all measures (rows). Human effect estimates are represented with black points for the mean, with shaded regions representing 95% credible intervals. Empirical null effects are denoted with lighter shading and an X on the mean estimate point. Predicted effect sizes from the best LM within each architecture (measured by goodness-of-fit to the filler sentences), and are sorted from largest to smallest, with error bars denoting 95% credible intervals.

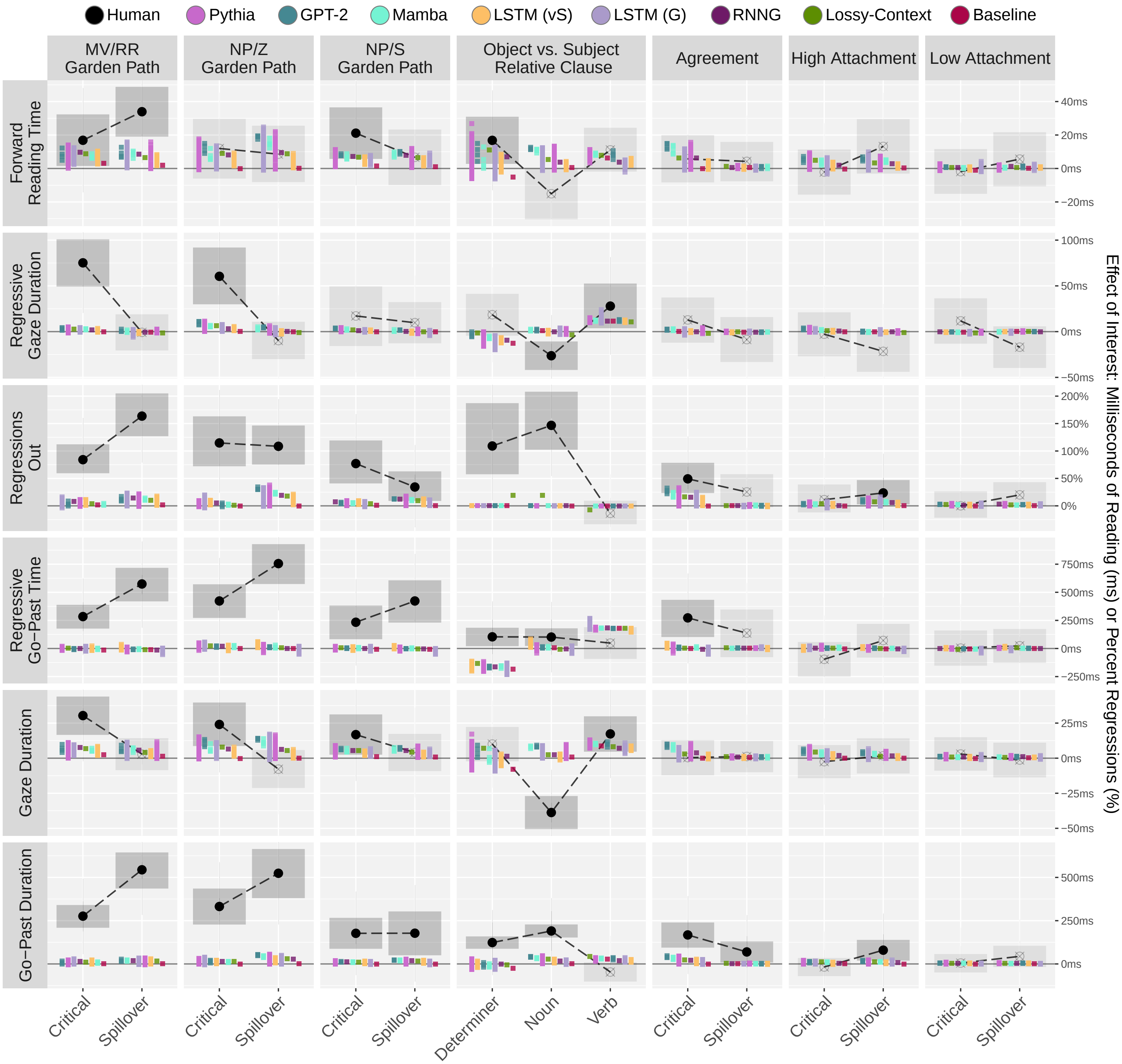

Fig. S4. Empirical (human) and predicted (LM) effects of interest at the regions of interest in all constructions (columns) and all measures (rows). Human effect estimates are represented with black points for the mean, with shaded regions representing 95% credible intervals. Empirical null effects are denoted with lighter shading and an X on the mean estimate point. Point estimates for the mean predicted effect size for all 409 LMs are plotted as square points, grouped by family, giving the full range of effect estimates for each LM family.

in favor of the alternative can be interpreted as "anecdotal" for a Bayes factor between 1–3, "moderate" for a Bayes factor between 3–10, "strong" for a Bayes factor between 10–30, and "very strong" for a Bayes factor of 30+. The same interpretation holds for the reciprocal of these cutoffs, but in favor of the null.

Results from the garden path constructions are summarized in Figure S5, and the other constructions are summarized in Figure S6.

In Forward Reading Time, Bayes factors consistently supported the equality of the surprisal-predicted and empirical aggregate garden path effects at both the critical and the spillover word. Evidence was "strong" for the null for the vaguest alternative (σ = 1*RMSE = 114 ms) and the second vaguest alternative (σ = 0.8*RMSE = 91 ms) at both regions, and was "strong" for the third vaguest alternative (σ = 0.5*RMSE = 57 ms) at the spillover region. For the no surprisal baseline models, the Bayes factors consistently supported the inequality of the surprisal-predicted and empirical effect sizes in Forward Reading Time.

In Regressive Gaze Duration, there was overwhelming evidence in favor the inequality of the surprisal-predicted and empirical effects for all regions of all constructions except for the verb region of the relative clause construction, where the Bayes factors favor the null for vaguer alternative sizes. However, it's important to note that results were almost identical for the baseline model, meaning the equality of the effects can be explained with baseline factors alone (in this case, it's likely that differences in word position may drive the effect).

In Regressions Out, evidence consistently favored the inequality of the empirical and surprisal-predicted effects, with the possible exception in the classic garden path subset of the spillover region in the NP/S construction. There is, however, evidence in favor of equality at the critical word in the agreement subset at the critical word, and in the high attachment condition of the attachment ambiguity subset at the spillover word. While there appears to be evidence in favor of equality at the verb region in the relative clause subset, it is important to note that the results are identical for the baseline model, meaning the apparent equality of the empirical and predicted effects is not driven by surprisal.

In Regressive Go-Past, evidence consistently favors the inequality of the empirical and surprisal-predicted effects for all constructions at all regions of interest, with the apparent exception of the noun region of the relative clause subset. However, the results were identical for the baseline model, meaning the apparent equality of the empirical and predicted effects is not driven by surprisal.

6. Detailed model results for "*No monotonic function of surprisal can simultaneously explain processing difficulty in syntactically challenging and simple sentences*"

Results for the LM in each model family with the highest ΔLL are summarized in Figure S7. Confidence intervals for condition mean estimates were computed using the `emmeans` R package on a linear mixed effects regression model fit to all reading data in each surprisal bin, with the following formula:

$$Measure \sim ambig + (1 \mid item)$$

To ensure that differences between filler and garden path reading times was not driven by differences in other baseline factors (such as word length and frequency), we also examine mean *residualized* reading measurements, where the residual reading times are calculated as the difference between an empirical reading time, and the predicted reading time from the baseline filler models. Results on residualized reading times are summarized in Figure S8. We find that the same qualitative patterns hold for both the raw and residualized reading data.

7. Detailed methods for "*LM surprisal explains forward reading but not rereading*"

A. Estimating effects due to syntactic disambiguation.

A.1. Empirical effects from human data. The empirical effects were estimated as the difference in each reading measure at each word region of interest between the conditions in each subset. To this end, Bayesian mixed-effects regression models were fit separately for each combination of subset, reading measure, and word region. Full model formulae can be found in Appendix B.

For the classic garden path subset, ambiguity was coded using contrast coding (unambiguous: 0, ambiguous: 1). The three constructions were coded using treatment coding, where the first variable `MVRR_vs_NPS` contrasted the MV/RR construction with the NP/S construction (MV/RR: 0, NP/S: 1), and the second variable `MVRR_vs_NPZ` contrasted the MV/RR construction with the NP/Z construction (MV/RR: 0, NP/Z: 1). The regression models included the main effects of ambiguity, `MVRR_vs_NPS`, and `MVRR_vs_NPZ`, as well as the two-way interactions `Ambiguity:MVRR_vs_NPS` and `Ambiguity:MVRR_vs_NPZ`. The models also included by-participant and by-item random slopes for all main and interaction effects, as well as by-participant and by-item random intercepts.

For the relative clause subset, clause type was coded using contrast coding (SRC: 0, ORC: 1). As the three word regions of interest (i.e., the determiner, noun, and verb) occurred at different linear positions across conditions, word position was first residualized out of each reading measure using a linear mixed-effects model fit to the filler sentences that includes the main effect of position, its by-participant random effect, and a by-participant random intercept. Subsequently, regression models that included the main effect of clause type, by-participant and by-item random slopes for clause type, and by-item random intercepts were fit to the residualized reading measures from each word region.

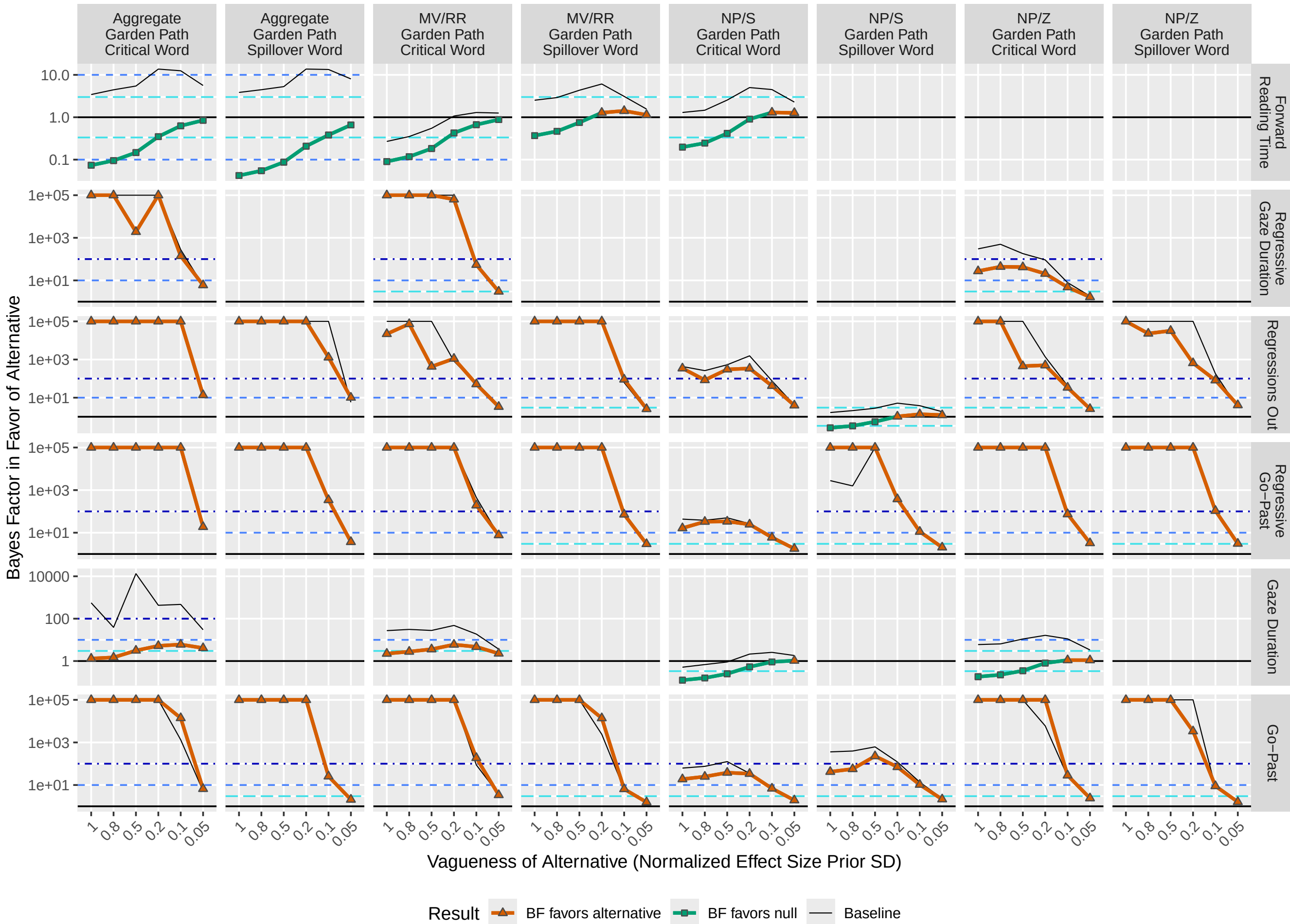

Fig. S5. Bayes factors (y-axis) for the classic garden path constructions at the critical and spillover regions (columns) for each measure (rows) using predicted RTs from the LM whose mean effect estimates were closest on average to the empirical effect estimates. Bayes factors greater than one (orange) favor the alternative (that the LM surprisal-predicted and empirical effects come from different distributions) and Bayes factors less than one (blue) favor the null (LM surprisal-predicted and empirical effects have the same distribution). Within each plot, Bayes factors range from the vaguest alternative on the left to the strictest alternative on the right. We include reference lines indicating "moderate" evidence in cyan, "strong" evidence in blue, and "extreme" evidence in dark blue. Plots are left empty if there was originally no evidence for an empirical effect for a particular construction-measure combination. Bayes factors for the baseline no-surprisal model's predictions are also provided for comparison (black). Note that support for the null hypothesis should only be attributed to surprisal if the baseline model favors the alternative instead.

For the attachment ambiguity subset, ambiguity and attachment location were coded using sum coding (unambiguous: $-\frac{1}{3}$, ambiguous: $\frac{2}{3}$; low: $-\frac{1}{2}$, high: $\frac{1}{2}$). The regression models included the main effects of ambiguity and attachment location, by-participant and by-item random slopes for both main effects, and by-participant and by-item random intercepts.

Finally, for the agreement violation subset, violation was coded using contrast coding (no violation: 0, violation: 1). The regression models included the main effect of violation, by-participant and by-item random slopes for violation, and by-participant and by-item random intercepts.

All regression models were fit using the `brms` package in R (37). For the reading time measures (all measures except Regressions Out), we use the same priors as (32). For Regressions Out, we set the prior on the intercept to $\mathcal{N}(-1.5, 1)$, the coefficients to $\mathcal{N}(0, 1)$, and the standard deviation of random effects to $\mathcal{N}_+(0, 1.5)$. Subsequently, four independent Markov Chain Monte Carlo chains were used to draw 12,000 samples each from the posterior distribution. The first half of the samples were treated as warmup samples and discarded, resulting in a total of 24,000 post-warmup draws across the four chains. The $\hat{R}$ value of all estimated effects were 1.00, which indicates model convergence.

A.2. Predicted effects from LM surprisal. The reading measures predicted by LM surprisal were estimated using protocols established in previous work for self-paced reading (32, 38, 39), adapted for eye-tracking data. First, for each combination of LM and reading measure, we fit a frequentist (non-Bayesian) mixed-effects regression model to data collected using the filler items, using the `lme4` package in R (37). These *filler models* include LM surprisal, word frequency, word length, and position of the word within the sentence as predictors. All predictors were scaled such that their mean was zero and their standard deviation was one. To account for spillover effects, the filler models also include the surprisal, frequency and length of the previous word (where surprisal was estimated by the same LM). They also included random slopes for surprisal and frequency by participant,

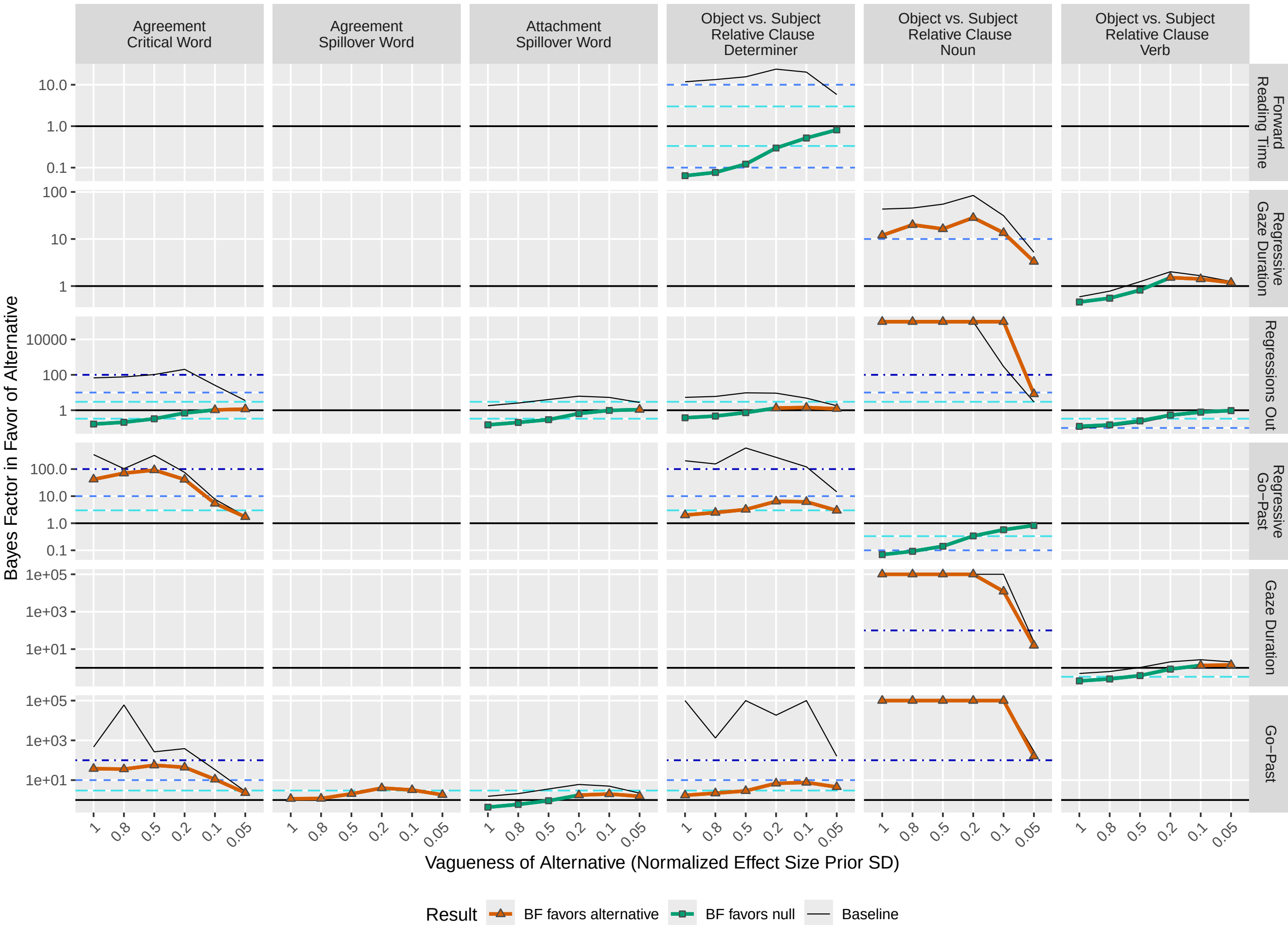

Fig. S6. Bayes factors (y-axis) for the relative clause, agreement, and attachment ambiguity subsets at the critical and spillover regions (columns) for each measure (rows) using predicted RTs from the LM whose mean effect estimates were closest on average to the empirical effect estimates. Bayes factors greater than one (orange) favor the alternative (that the LM surprisal-predicted and empirical effects come from different distributions) and Bayes factors less than one (blue) favor the null (LM surprisal-predicted and empirical effects have the same distribution). Bayes factors for the baseline no-surprisal model's predictions are also provided for comparison (black). Within each plot, Bayes factors range from the vaguest alternative on the left to the strictest alternative on the right. We include reference lines indicating "moderate" evidence in cyan, "strong" evidence in blue, and "extreme" evidence in dark blue. Plots are left empty if there was originally no evidence for an empirical effect for a particular construction-measure combination. Note that support for the null hypothesis should only be attributed to surprisal if the baseline model favors the alternative instead.

and random intercepts for both participants and items. Sentence-final words were excluded from the filler data, following (32). The full formulae for the baseline and surprisal filler models can be found in Appendix B. For filler models predicting Regressions Out, a binary response variable, we use a logit link; for all other filler models, where the predicted measure is continuous, we use an identity link. Subsequently, these filler models were used to calculate the predicted trial-level reading measures for the words of interest in each condition. Finally, the difference in the predicted reading measures across conditions was estimated by fitting regression models with identical specifications to those that were used to estimate empirical effects. For Regressions Out, the filler model-predicted reading data was expressed as log-odds of a regression, and the experimental effects were estimated on the logits using linear regression.

B. Regression model formulae. Model formulae are provided in R formula notation. The variable to the left of the "$\sim$" symbol is the response variable, and all variables to its right are predictors. The "$a \cdot b$" symbol denotes that a and b are combined both additively and multiplicitively. Random effects are denoted with "$\|$" for uncorrelated random intercepts and slopes, and "$|$" for correlated random slopes. The random effect structure appears to the left of the "$|$" or "$\|$" symbol, and the grouping variable appears to the right. Random intercepts are denoted with "1", and random slopes are denoted with their variable name. "$Surp(w_i)$ denotes the surprisal of the ith word of a sentence. Omitted from the formula below is the fact that all predictors are scaled to a mean of zero and a standard deviation of one (predictions on the syntactically challenging sentences are scaled based on the means and standard deviations from the filler data). For the upper-bound filler models, $Measure_B$ denotes a reading measure from only participants in subset B, and $\overline{Measure_A}(w_i)$ is the mean of this measure at the ith word of the sentence across all participants in subset A.

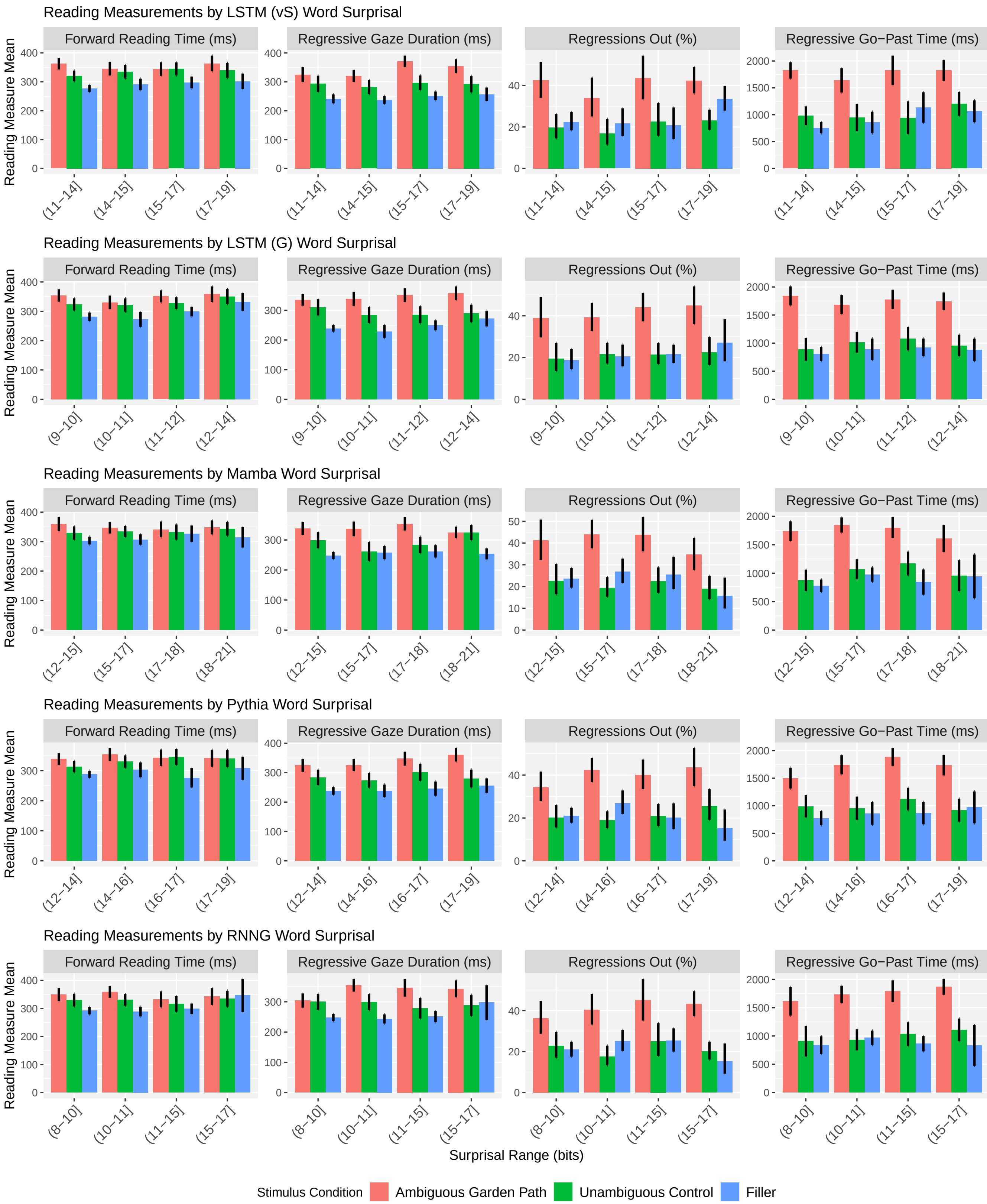

Fig. S7. Mean reading times/regression rates for the critical/spillover word of ambiguous garden path sentences (red), binned by the surprisal assigned to that word in the LM within each family with the highest ΔLL, along with item-matched unambiguous control sentences (green), and surprisal-matched filler words (blue). Reading times are measured at the word with the stronger relationship to surprisal in the filler models: the current word for FORWARD READING TIME and REGRESSIVE GAZE or the following word for REGRESSIONS OUT and REGRESSIVE GO-PAST. Error bars represent 95% confidence intervals adjusted for repeated measures.

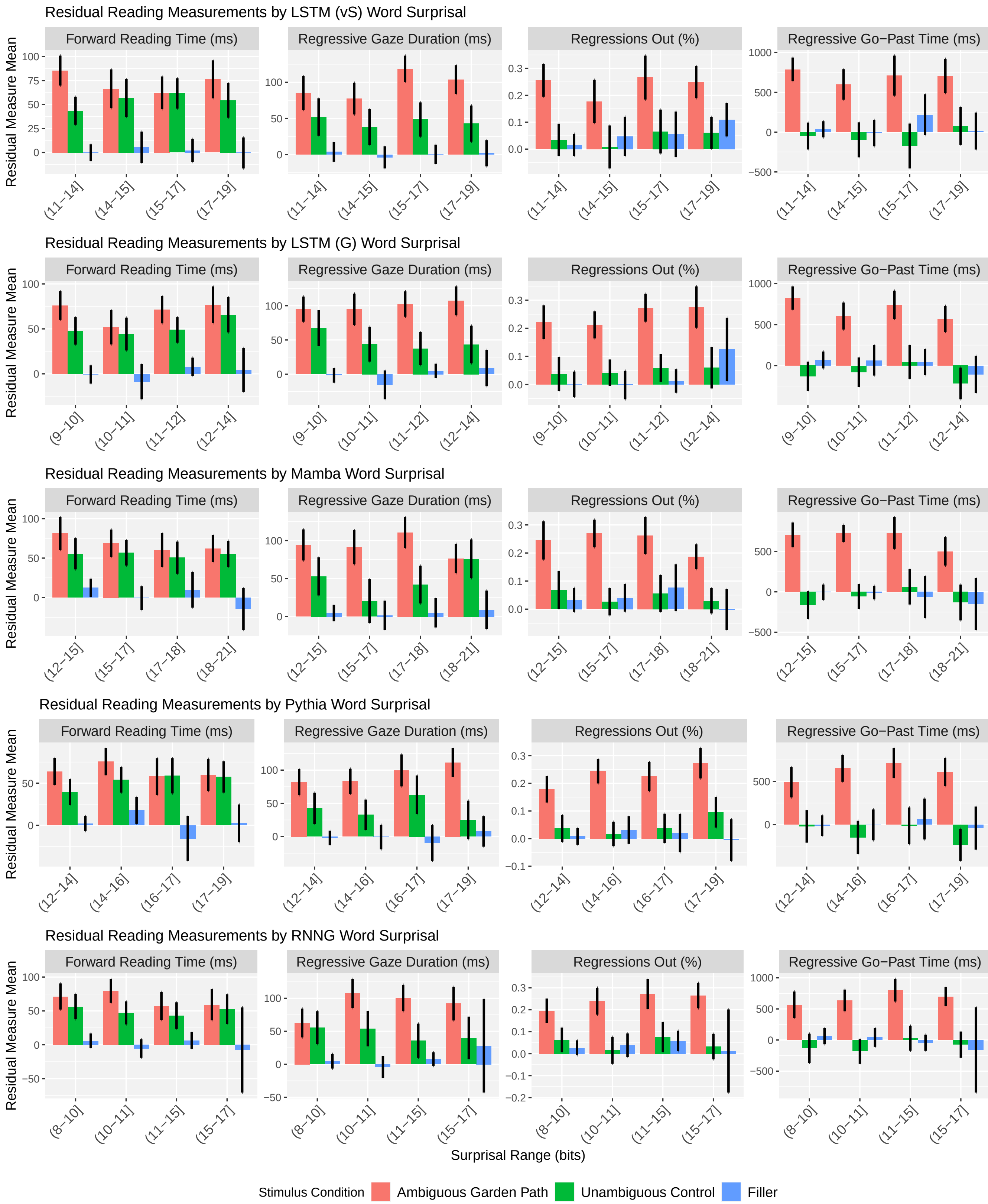

Fig. S8. Residualized mean reading times/regression rates for the critical/spillover word of ambiguous garden path sentences (red), binned by the surprisal assigned to that word in the LM within each family with the highest ΔLL, along with item-matched unambiguous control sentences (green), and surprisal-matched filler words (blue). Reading times are measured at the word with the stronger relationship to surprisal in the filler models: the current word for FORWARD READING TIME and REGRESSIVE GAZE or the following word for REGRESSIONS OUT and REGRESSIVE GO-PAST. Error bars represent 95% confidence intervals adjusted for repeated measures.

Full filler model formula:

$$\begin{aligned} Measure \sim\; & Surp(w_i) + Surp(w_{i-1}) + \\ & \big(Logfreq(w_i) \cdot Length(w_i)\big) + \big(Logfreq(w_{i-1}) \cdot Length(w_{i-1})\big) + \\ & Position(w_i) + \\ & \big(1 + Surp(w_i) + Surp(w_{i-1}) + Logfreq(w_i) + Logfreq(w_{i-1}) \parallel participant\big) + \\ & (1 \mid item) \end{aligned}$$

Baseline filler model formula:

$$\begin{aligned} Measure \sim\; & \big(Logfreq(w_i) \cdot Length(w_i)\big) + \big(Logfreq(w_{i-1}) \cdot Length(w_{i-1})\big) + \\ & Position(w_i) + \\ & \big(1 + Logfreq(w_i) + Logfreq(w_{i-1}) \parallel participant\big) + \\ & (1 \mid item) \end{aligned}$$

Upper-bound full filler model formula:

$$\begin{aligned} Measure_B \sim\; & \overline{Measure_A}(w_i) + \overline{Measure_A}(w_{i-1}) + \\ & \big(Logfreq(w_i) \cdot Length(w_i)\big) + \big(Logfreq(w_{i-1}) \cdot Length(w_{i-1})\big) + \\ & Position(w_i) + \\ & \big(1 + \overline{Measure_A}(w_i) + \overline{Measure_A}(w_{i-1}) + \\ & \; Logfreq(w_i) + Logfreq(w_{i-1}) \parallel participant\big) + \\ & (1 \mid item) \end{aligned}$$

Upper-bound baseline filler model formula:

$$\begin{aligned} Measure_B \sim\; & \big(Logfreq(w_i) \cdot Length(w_i)\big) + \big(Logfreq(w_{i-1}) \cdot Length(w_{i-1})\big) + \\ & Position(w_i) + \\ & \big(1 + Logfreq(w_i) + Logfreq(w_{i-1}) \parallel participant\big) + \\ & (1 \mid item) \end{aligned}$$

Agreement subset model formula:

$$\begin{aligned} Measure \sim\; & agree + \\ & (1 + agree \parallel subject) + \\ & (1 + agree \parallel item) \end{aligned}$$

Attachment ambiguity subset model formula:

$$\begin{aligned} Measure \sim\; & ambiguity + height + \\ & (1 + ambiguity + height \parallel subject) + \\ & (1 + ambiguity + height \parallel item) \end{aligned}$$

Classic garden path subset model formula:

$$\begin{aligned} Measure \sim\; & ambiguity \cdot (NPS + NPZ) + \\ & \big(1 + ambiguity \cdot (NPS + NPZ) \parallel subject\big) + \\ & \big(1 + ambiguity \cdot (NPS + NPZ) \parallel item\big) \end{aligned}$$

Relative clause subset word position residualization model formula:

$$\begin{aligned} Measure \sim\; & Position(w_i) + \\ & \big(1 + Position(w_i) \parallel subject\big) + \\ & \big(1 \mid item\big) \end{aligned}$$

Relative clause subset model formula:

$$Measure\_corrected \sim RC\_type + (0 + RC\_type \,||\, subject) + (1 + RC\_type \,||\, item)$$

8. Distributions of LM surprisal values

Histograms of LM surprisals assigned to filler and classic garden path sentences by the model from each LM family that produced the highest ΔLL (i.e., highest increase in the likelihood of the empirical data compared to a baseline model that did not include LM surprisal) are provided in Figure S9. In general, there is substantial overlap in the distribution of LM surprisal values in the filler sentences and the classic garden path sentences across all LM families. In all LM families except `LSTM (G)` there is at least one word in the filler sentences with a higher LM surprisal than any of the critical words in the classic garden path sentences. This means that the failure of LM surprisal to capture the magnitude of syntactic processing effects in the regressive measures cannot be explained by a difference in the distribution of LM surprisal values that the filler models are fit to, and those at the critical region of syntactically challenging sentences.

9. Influence of LM size and amount of training data on garden path effect magnitudes

The number of parameters (size) of the LMs we evaluated varied in four model families: `GPT-2`, `Mamba`, `LSTM (vS)`, and `Pythia`. The amount of training data only varied systematically in `LSTM (vS)` and `Pythia`. We evaluate the relationship between model size/training data size and the magnitude of predicted garden path effects through simple visual inspection of plots within model families. Garden path effects reported for each model were estimated using frequentist linear mixed effects models fit with the `lme4` package in `R` with default settings. The model formulae were identical in specification to the Bayesian models presented in the main body of the paper.

GPT-2 family. In all measures, there appears to be a negative correlation between model size and the magnitude of predicted GP effects, where larger garden path effects are predicted by the models with fewer parameters. Effects in all measures are summarized in Figure S10 (top).

Mamba family. In Forward Reading Time and Regressive Gaze, there appears to be a small negative correlation between model size and the magnitude of predicted GP effects, where larger garden path effects are predicted by the models with fewer parameters, but does not appear to hold in Regressions Out or Regressive Go-Past Time. Effects in all measures are summarized in Figure S10 (bottom).

LSTM (vS) family. In the LSTM (vS) models, it appears to be the case that for a fixed amount of training data, larger model sizes are weakly associated with larger predicted garden path effects in Regressions Out, Forward Reading Time, Regressive Gaze and perhaps to a lesser extent in Regressive Go-Past. The same general pattern also appears to hold for the amount of training data. Models of a given size that were trained on a larger corpus appear to predict larger garden path effects, though this relationship was less clear on visual inspection in Regressive Go-Past compared to the other measures. Effects in Regressions Out are summarized in Figure S11, Forward Reading Time in Figure S12, Regressive Gaze in Figure S13 and Regressive Go-Past in Figure S14.

Pythia family. Unlike the `LSTM (vS)` models, `Pythia` LMs with more parameters generally predicted smaller garden path effects for a given amount of training data, with the four smallest LMs generally predicting the largest garden path effects. Note however that even the smallest Pythia models are larger in their total parameter count than the largest `LSTM (vS)` models. Interestingly, it was not the case that larger LMs predicted larger garden path effects early in training and smaller LMs predicted larger garden path effects later in training. Instead, the smaller LMs generally predicted larger garden path effects both early and late in training. This trend appears to hold across all measures. As for the effect of training data, it appears that garden path effect magnitudes peak around 3,000–16,000 steps, and are consistently smaller than this peak in the models trained on the most data (143,000 steps). The effect of training data amount appears to be weaker in Regressions Out than the other measures. Effects in Regressions Out are summarized in Figure S15, Forward Reading Time in Figure S16, Regressive Gaze in Figure S17 and Regressive Go-Past in Figure S18.

Summary. In general, larger garden path effects were predicted by LMs with fewer parameters, but only beyond a certain point, and in models trained on a relatively modest amount of training data. In the relatively small `LSTM (vS)` models, larger model sizes and more training data predicted larger garden path effects across all measures, while in the `Mamba`, `GPT-2` and `Pythia` models, we generally saw the opposite pattern. Overall, our results are consistent with several works demonstrating that larger LMs trained on more data achieve lower perplexity (i.e., they are better at their training objective of predicting the next word), but provide a poorer fit to human reading times in naturalistic reading corpora (40–42). These results present a serious challenge to the view that the gap between LM-surprisal-based predictions and human reading behavior can simply be bridged through improvements to LMs on their training objective alone (43, 44).

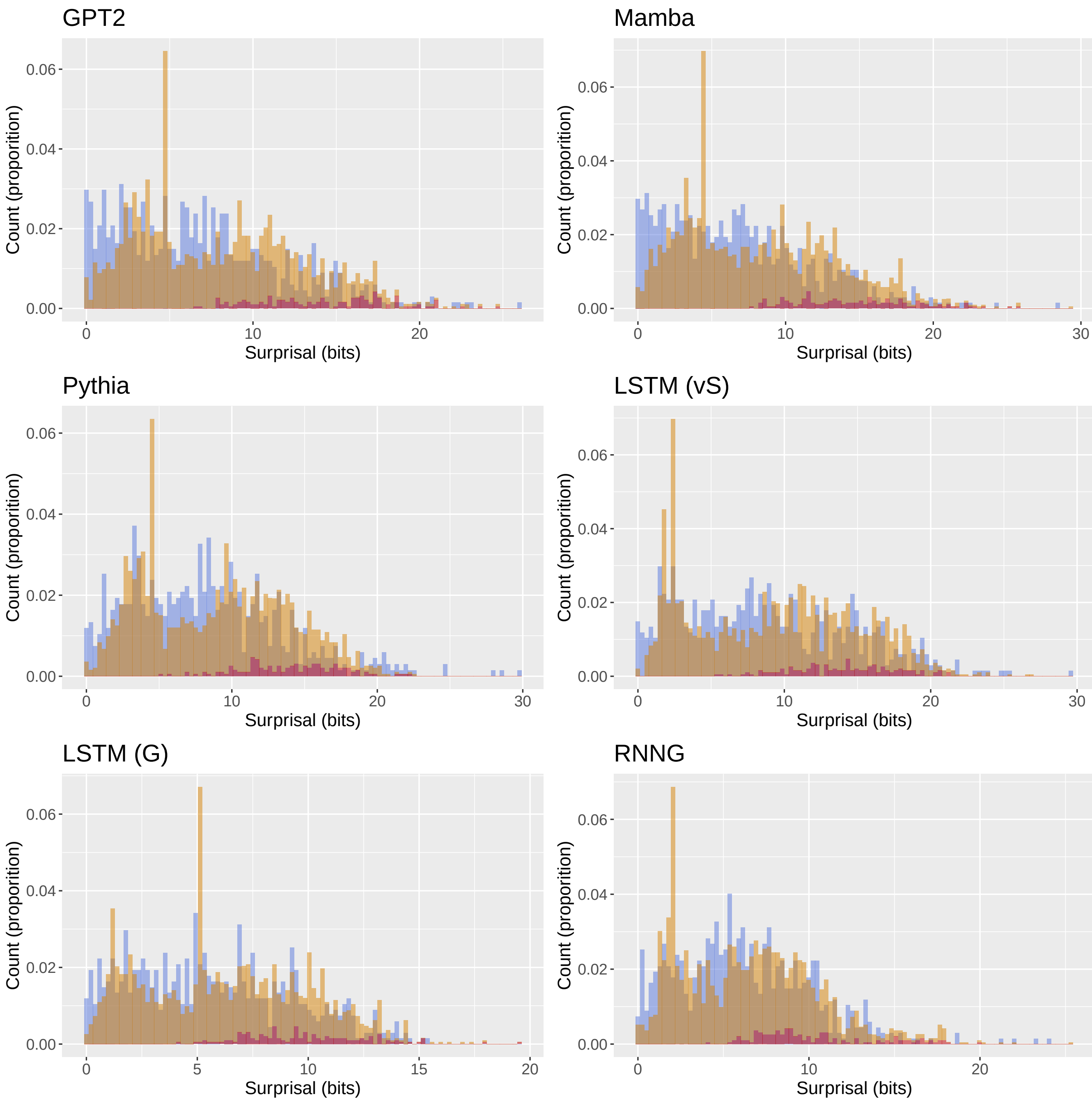

Fig. S9. Distribution of LM surprisal values assigned to filler words (blue) and classic garden path sentence words, where words in the critical region are colored red, and words outside the critical region are colored orange.

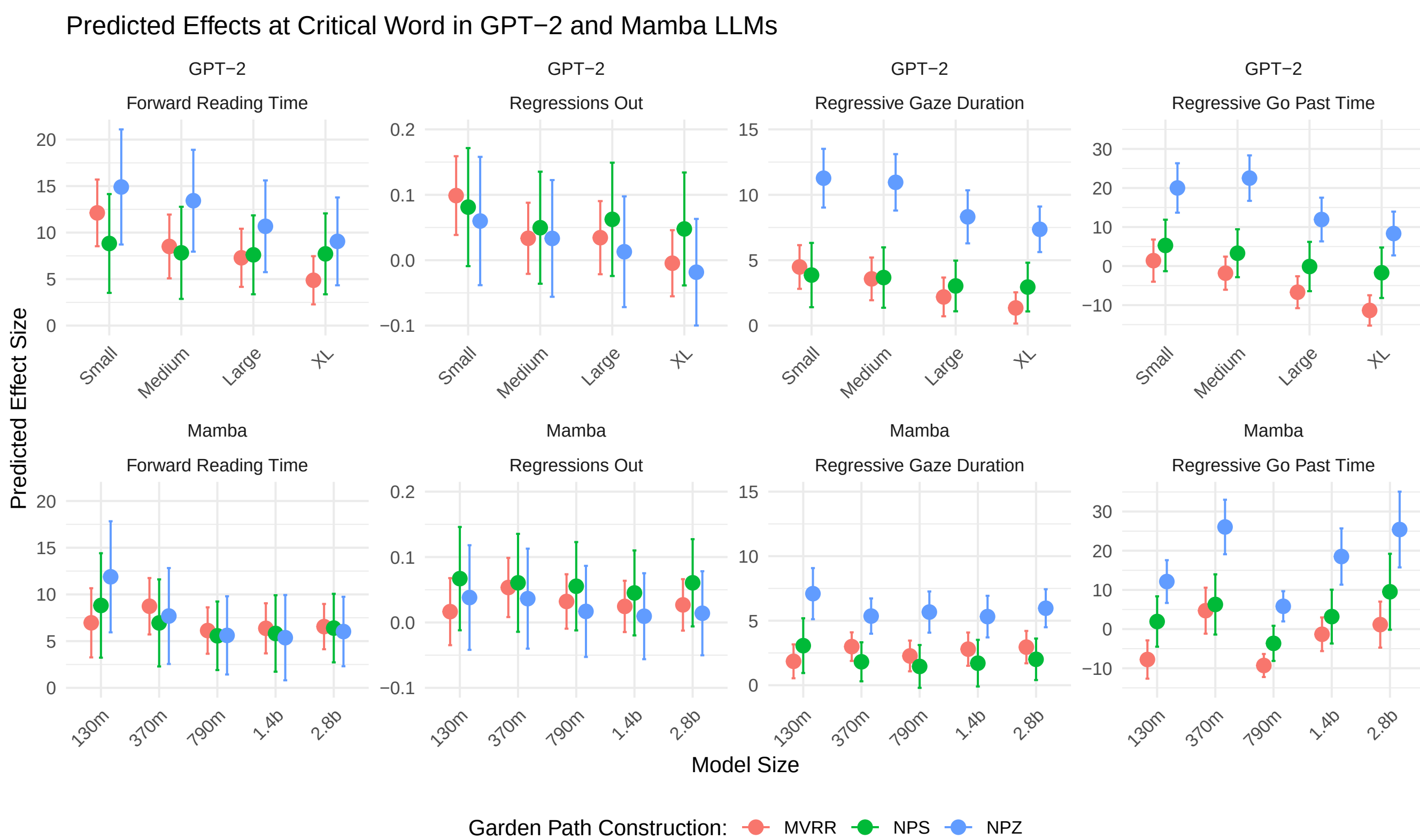

Fig. S10. Predicted garden path effects in all four measures (columns) from `GPT-2` (top) and `Mamba` (bottom) LM families. Effects in REGRESSIONS OUT are measured as the difference in log-odds of a regression between the ambiguous and unambiguous conditions. All other effects are measured as the difference in milliseconds of predicted reading time between conditions. Within each plot, models are arranged from smallest to largest in parameter count.

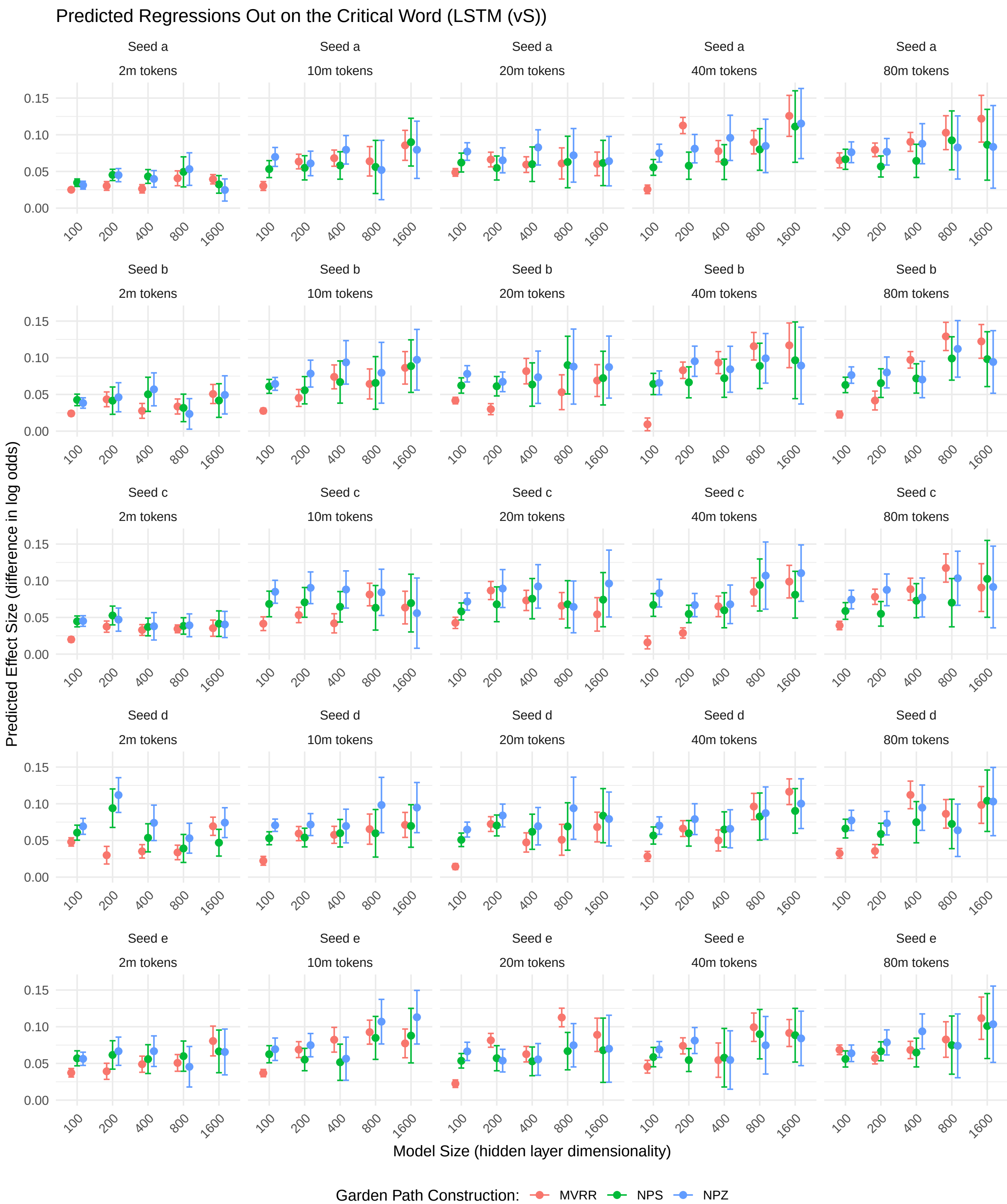

Fig. S11. `LSTM (vS)` predicted garden path effects in REGRESSIONS OUT, measured as the difference in log-odds of a regression between the ambiguous and unambiguous conditions. Models are grouped by random seed (the initial parameter weights and training data partition) and amount of training data. Within each plot, the x-axis denotes the model size (number of hidden units in each of the LM's two hidden layers), and the y-axis denotes the magnitude of the predicted garden path effect, with bootstrapped 95% confidence intervals.

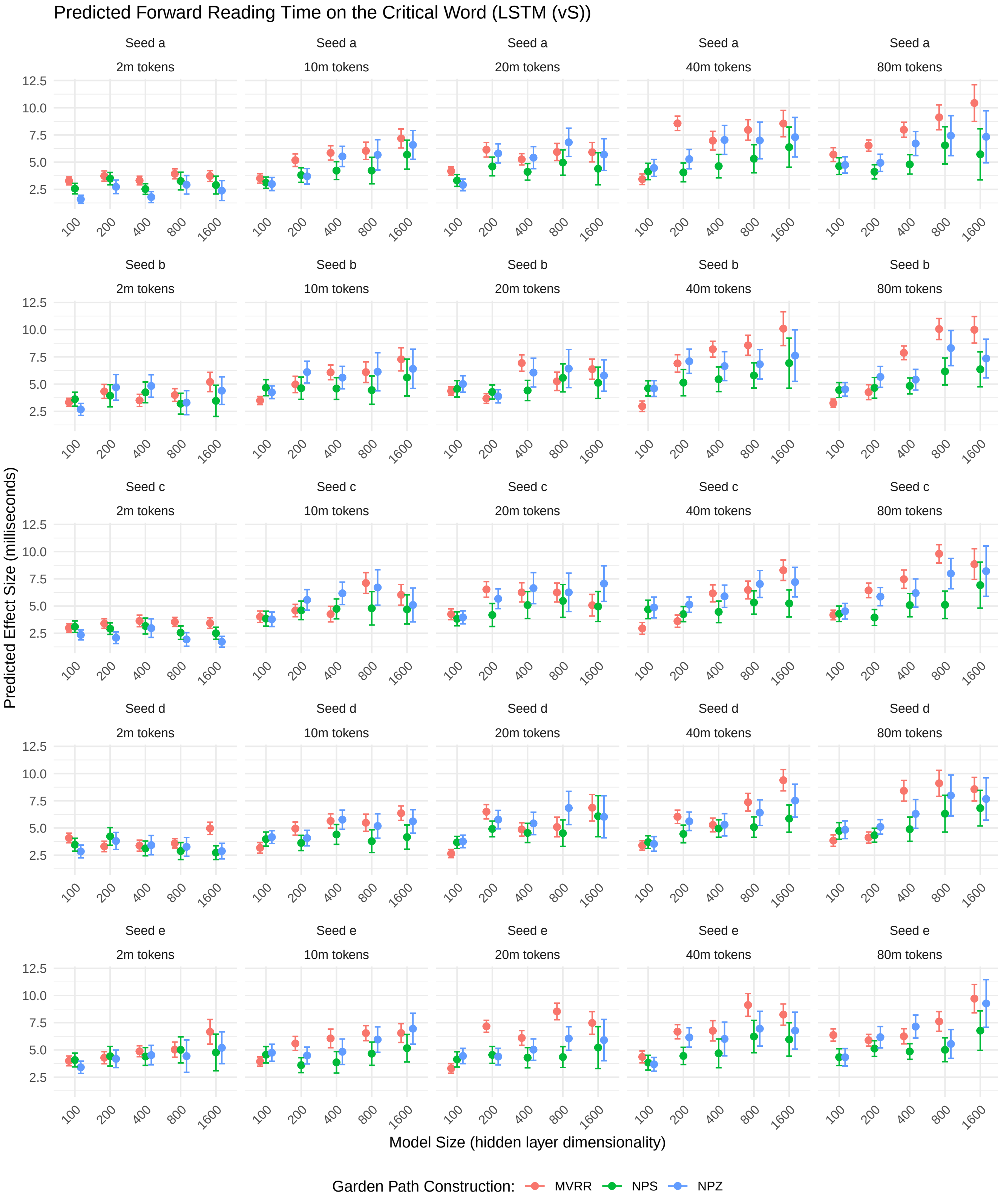

Fig. S12. `LSTM (vS)` predicted garden path effects in FORWARD READING TIME, measured as the difference in reading time between the ambiguous and unambiguous conditions. Models are grouped by random seed (the initial parameter weights and training data partition) and amount of training data. Within each plot, the x-axis denotes the model size (number of dimensions in each of the LM's two hidden layers), and the y-axis denotes the magnitude of the predicted garden path effect, with bootstrapped 95% confidence intervals.

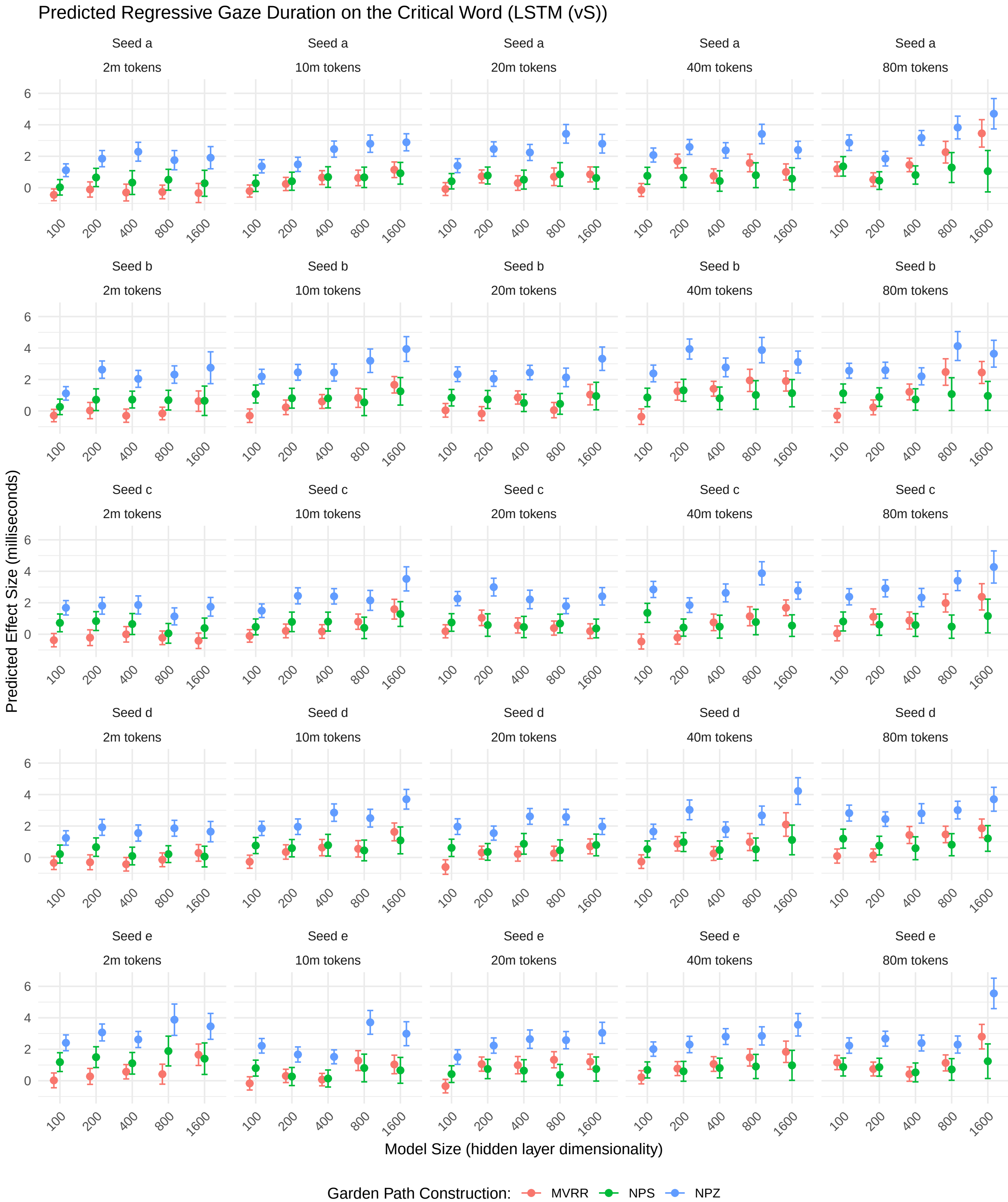

Fig. S13. `LSTM (vS)` predicted garden path effects in Regressive Gaze, measured as the difference in reading time between the ambiguous and unambiguous conditions. Models are grouped by random seed (the initial parameter weights and training data partition) and amount of training data. Within each plot, the x-axis denotes the model size (number of dimensions in each of the LM's two hidden layers), and the y-axis denotes the magnitude of the predicted garden path effect, with bootstrapped 95% confidence intervals.

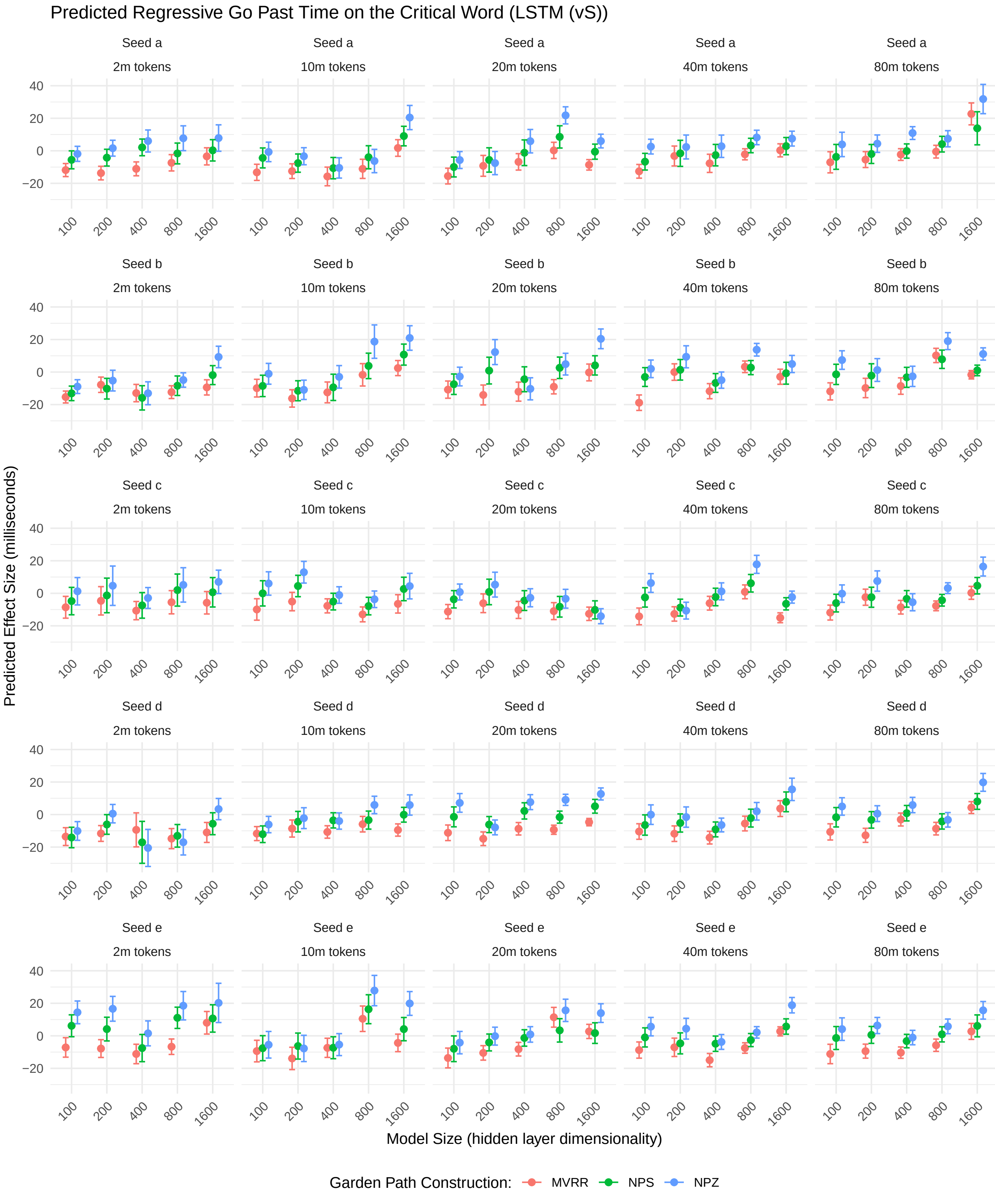

Fig. S14. `LSTM (vS)` predicted garden path effects in REGRESSIVE GO-PAST, measured as the difference in reading time between the ambiguous and unambiguous conditions. Models are grouped by random seed (the initial parameter weights and training data partition) and amount of training data. Within each plot, the x-axis denotes the model size (number of dimensions in each of the LM's two hidden layers), and the y-axis denotes the magnitude of the predicted garden path effect, with bootstrapped 95% confidence intervals.

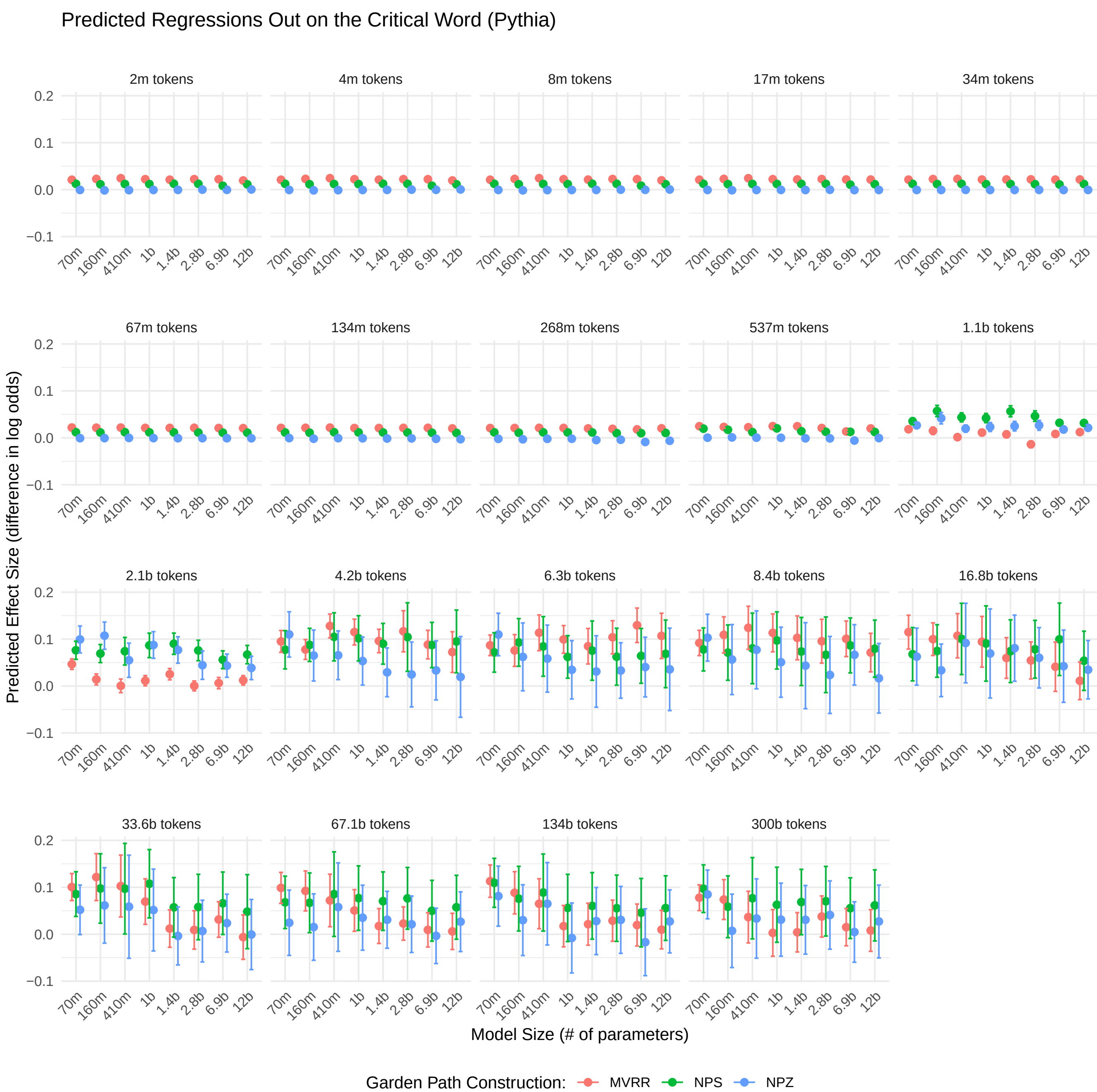

Fig. S15. `Pythia` predicted garden path effects in REGRESSIONS OUT, measured as the difference in log-odds of a regression between the ambiguous and unambiguous conditions. Plots are grouped by the LM's training data quantity. Within each plot, the x-axis denotes LM size (total number of parameters), the y-axis denotes the magnitude of the predicted garden path effect, with bootstrapped 95% confidence intervals.

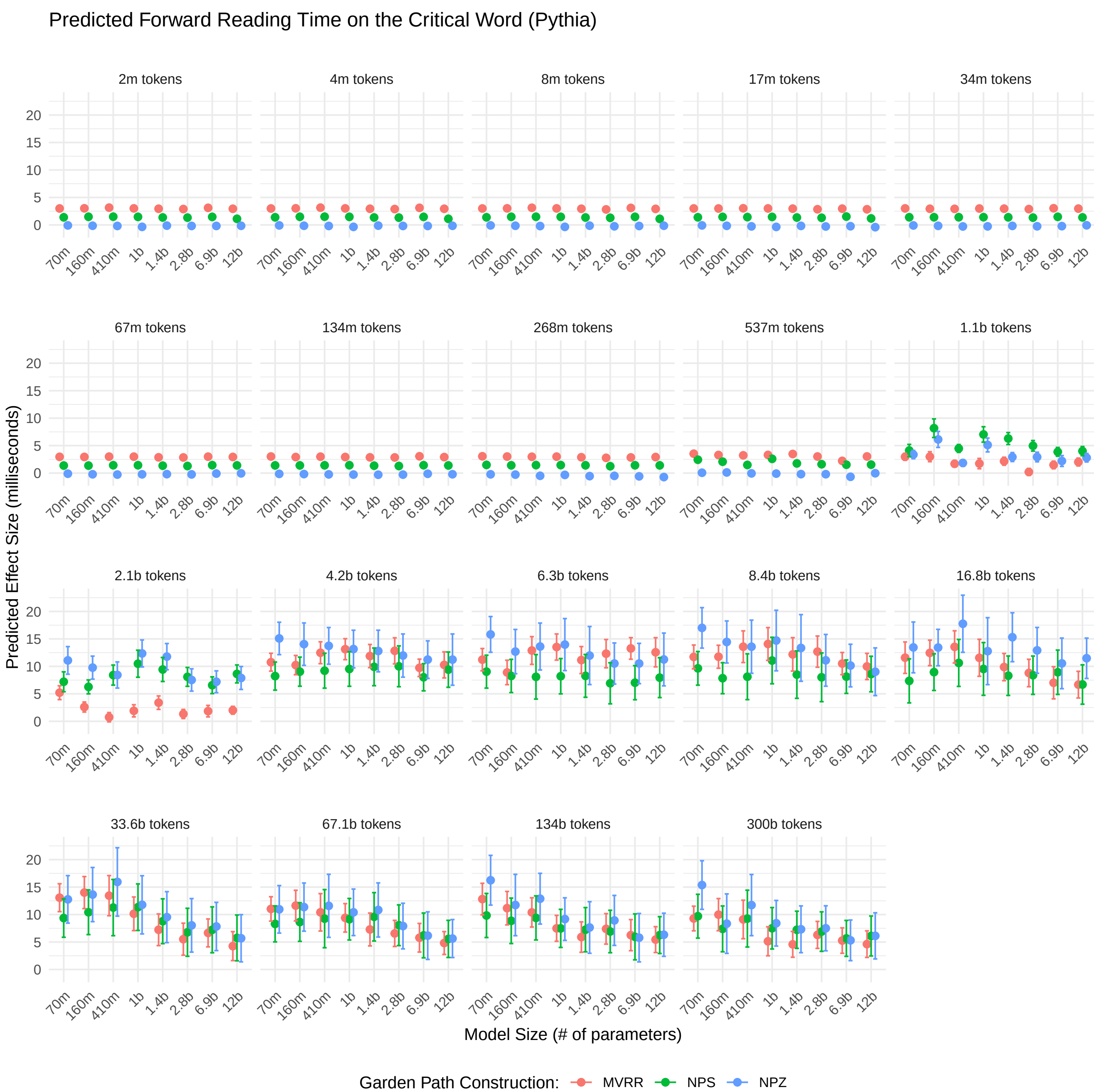

Fig. S16. `Pythia` predicted garden path effects in FORWARD READING TIME, measured as the difference in reading time between the ambiguous and unambiguous conditions. Plots are grouped by the LM's training data quantity. Within each plot, the x-axis denotes LM size (total number of parameters), the y-axis denotes the magnitude of the predicted garden path effect, with bootstrapped 95% confidence intervals.

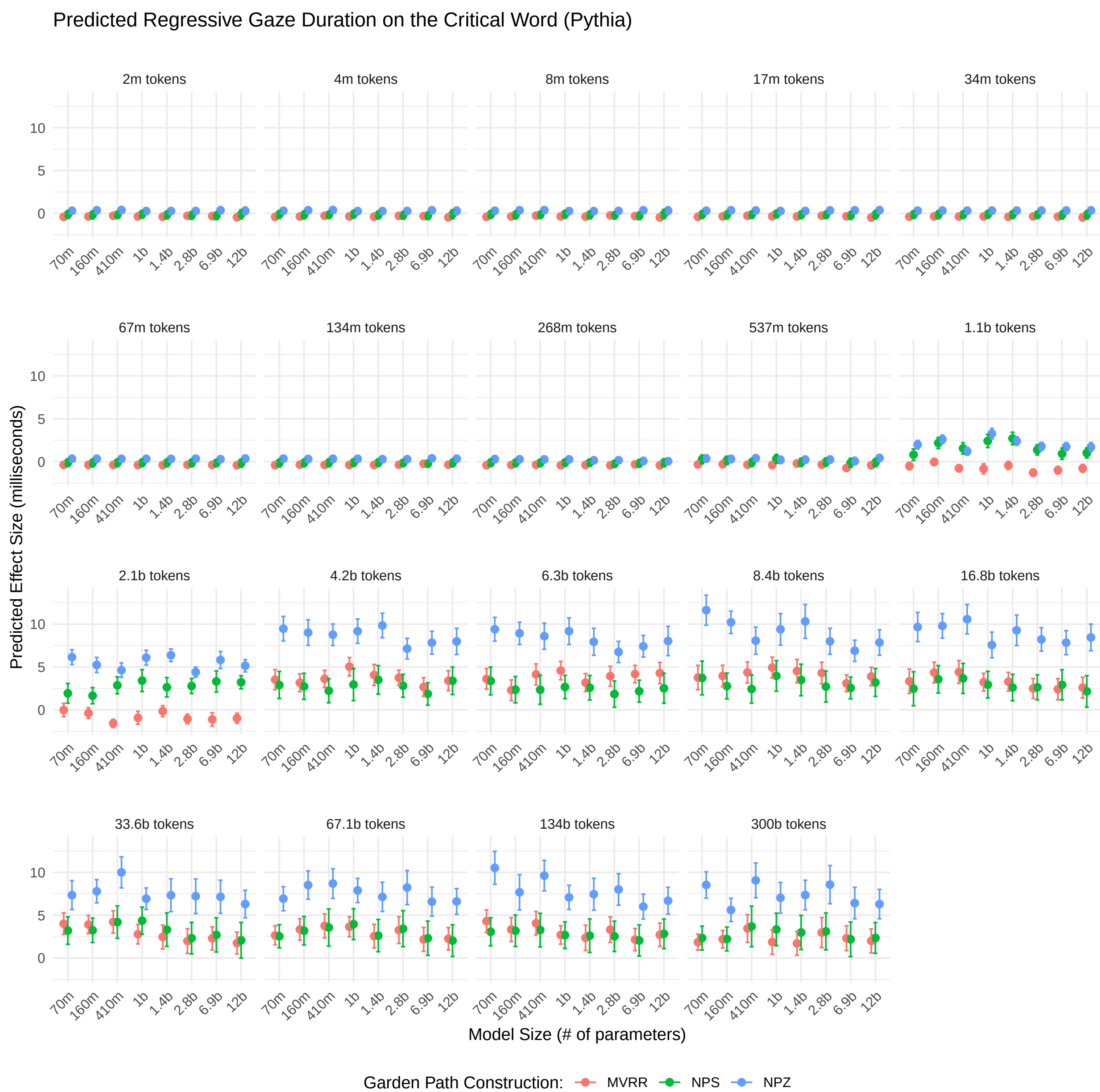

Fig. S17. `Pythia` predicted garden path effects in REGRESSIVE GAZE, measured as the difference in reading time between the ambiguous and unambiguous conditions. Plots are grouped by the LM's training data quantity. Within each plot, the x-axis denotes LM size (total number of parameters), the y-axis denotes the magnitude of the predicted garden path effect, with bootstrapped 95% confidence intervals.

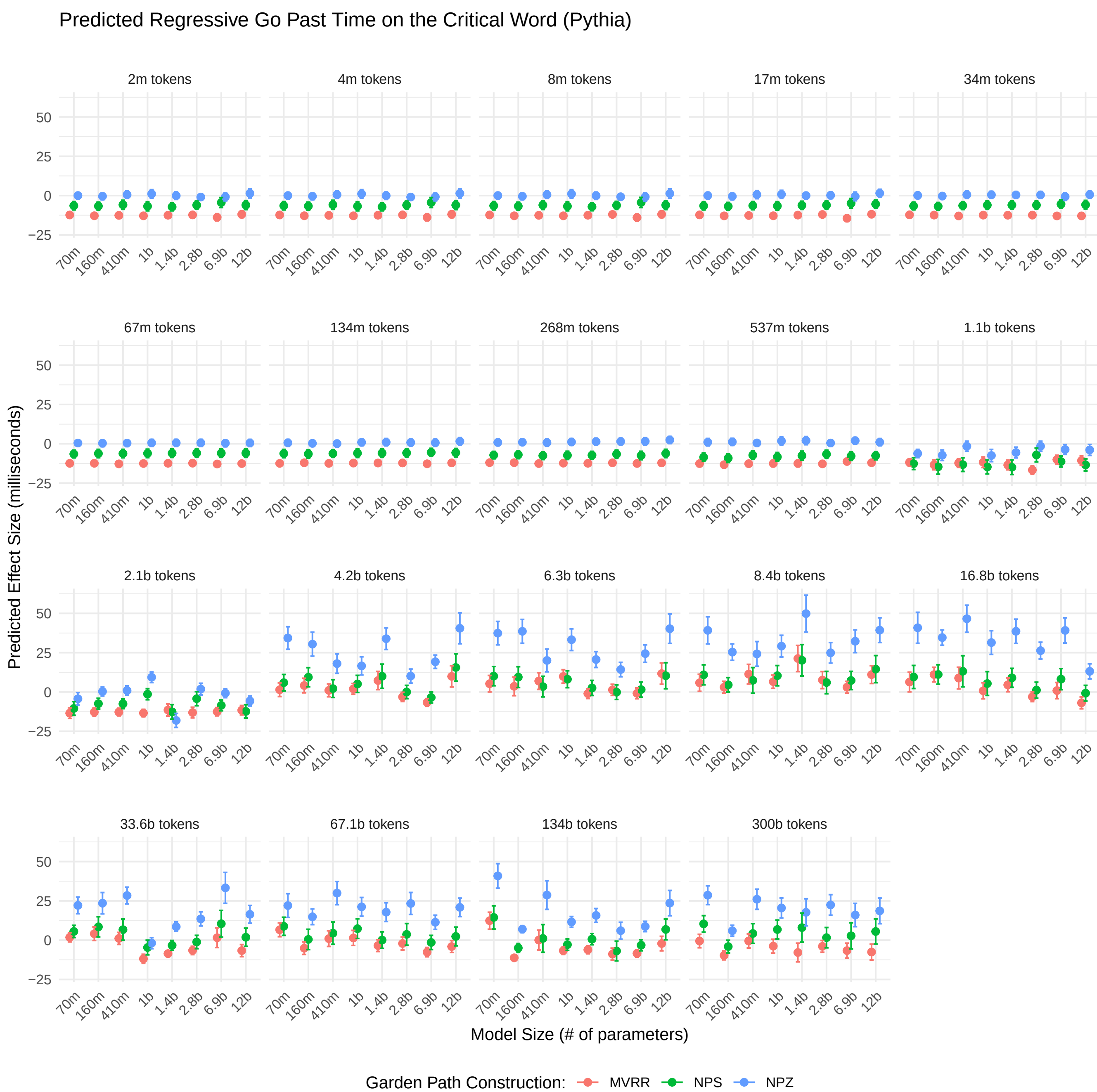

Fig. S18. `Pythia` predicted garden path effects in REGRESSIVE GO-PAST, measured as the difference in reading time between the ambiguous and unambiguous conditions. Plots are grouped by the LM's training data quantity. Within each plot, the x-axis denotes LM size (total number of parameters), the y-axis denotes the magnitude of the predicted garden path effect, with bootstrapped 95% confidence intervals.

10. Full list of experimental sentences

This section provides a complete list of the experimental items in each subset.

A. Classic garden path subset. For the first item, we fully spell out all six conditions. From the second item onward, we use a more concise notation, where the unambiguous conditions are differentiated by the material in parentheses.

1. (a) **Main Verb/Reduced Relative (MV/RR)**
   i. **Ambiguous**: The suspect sent the file deserved further investigation after the murder trial.
   ii. **Unambiguous**: The suspect who was sent the file deserved further investigation after the murder trial.

   (b) **Transitive/Intransitive (NP/Z)**
   i. **Ambiguous**: Because the suspect changed the file deserved further investigation after the murder trial.
   ii. **Unambiguous**: Because the suspect changed, the file deserved further investigation after the murder trial.

   (c) **Direct Object/Sentential Complement (NP/S)**
   i. **Ambiguous**: The suspect showed the file deserved further investigation after the murder trial.
   ii. **Unambiguous**: The suspect showed that the file deserved further investigation after the murder trial.

2. (a) The corrupt politician (who was) handed the bill received unwelcome attention from southern voters.
   (b) After the corrupt politician signed(,) the bill received unwelcome attention from southern voters.
   (c) The corrupt politician mentioned (that) the bill received unwelcome attention from southern voters.

3. (a) The woman (who was) brought the mail disappeared mysteriously after reading the bad news in it.
   (b) After the woman moved(,) the mail disappeared mysteriously from the delivery system.
   (c) The woman maintained (that) the mail disappeared mysteriously from her front porch.

4. (a) The boy (who was) fed the chicken stayed surprisingly happy despite having a mild allergic reaction.
   (b) Although the boy attacked(,) the chicken stayed surprisingly happy as if nothing happened.
   (c) The boy found (that) the chicken stayed surprisingly happy in the new barn.

5. (a) The new doctor (who was) offered the operation appeared increasingly likely to succeed in her career.
   (b) After the new doctor left(,) the operation appeared increasingly likely to succeed.
   (c) The new doctor demonstrated (that) the operation appeared increasingly likely to succeed.

6. (a) The professor (who was) awarded the grant gained more attention from marine biologists.
   (b) After the professor read(,) the grant gained more attention due to her excellent description.
   (c) The professor noticed (that) the grant gained more attention from marine biologists.

7. (a) The technician (who was) refused the service stopped working almost immediately after the argument.
   (b) After the technician called(,) the service stopped working almost immediately to his surprise.
   (c) The technician reported (that) the service stopped working almost immediately after the storm started.

8. (a) The mechanic (who was) brought the truck needed several more hours to fully repair it.
   (b) Because the mechanic stopped(,) the truck needed several more hours before it could be fully repaired.
   (c) The mechanic observed (that) the truck needed several more hours to be repaired.

9. (a) The guitarist (who was) assigned the song failed dramatically because he never practiced enough.
   (b) After the guitarist began(,) the song failed dramatically because he skipped the sound check.
   (c) The guitarist knew (that) the song failed dramatically because of the tensions within the band.

10. (a) The player (who was) paid the bonus remained essentially the same despite his sudden fame and wealth.
    (b) Although the player lost(,) the bonus remained essentially the same as in the original contract.
    (c) The player revealed (that) the bonus remained essentially the same as in the original contract.

11. (a) The recent hire (who was) offered the job prepared many students for careers in media.
    (b) Once the recent hire started(,) the job prepared many students for careers in media.
    (c) The recent hire claimed (that) the job prepared many students for careers in media.

12. (a) The assistant manager (who was) assigned the training seemed unnecessarily demanding to new staff.
    (b) While the assistant manager worked(,) the training seemed unnecessarily demanding to him.
    (c) The assistant manager discovered (that) the training seemed unnecessarily demanding for new staff.
13. (a) The mayor (who was) sent the document provided sufficient evidence that it was simply blackmail.
    (b) Although the mayor changed(,) the document provided sufficient evidence for what he had promised.
    (c) The mayor showed (that) the document provided sufficient evidence to prove her innocence.
14. (a) The basketball player (who was) handed the contract created another controversy in the NBA.
    (b) After the basketball player signed(,) the contract created another political controversy in the NBA.
    (c) The basketball player mentioned (that) the contract created another controversy in the NBA.
15. (a) The engineer (who was) brought the equipment required constant supervision from senior technicians.
    (b) After the engineer moved(,) the equipment required constant supervision from senior technicians.
    (c) The engineer maintained (that) the equipment required constant supervision from senior technicians.
16. (a) The little girl (who was) fed the lamb remained relatively calm despite having asked for beef.
    (b) When the little girl attacked(,) the lamb remained relatively calm despite the sudden assault.
    (c) The little girl found (that) the lamb remained relatively calm despite the absence of its mother.
17. (a) The yoga instructor (who was) offered the position demanded immense physical effort from everyone.
    (b) Before the yoga instructor left(,) the position demanded immense physical effort from everyone.
    (c) The yoga instructor demonstrated (that) the position demanded immense physical effort from everyone.
18. (a) The governor (who was) awarded the contract received sweeping support across the entire state.
    (b) While the governor read(,) the contract received sweeping support from the audience at the rally.
    (c) The governor noticed (that) the contract received sweeping support across the entire state.
19. (a) The patient (who was) refused the treatment continued causing uncomfortable scenes in the ER.
    (b) Before the patient called(,) the treatment continued causing uncomfortable side effects like nausea.
    (c) The patient reported (that) the treatment continued causing uncomfortable side effects like nausea.
20. (a) The operator (who was) brought the machine started working efficiently with the added automation.
    (b) Once the operator stopped(,) the machine started working efficiently without any supervision.
    (c) The operator observed (that) the machine started working efficiently all of a sudden.
21. (a) The dancer (who was) assigned the ballet achieved incredible success for a new performer.
    (b) Once the dancer began(,) the ballet achieved incredible success for a show with a new performer.
    (c) The dancer knew (that) the ballet achieved incredible success for a small local production.
22. (a) The contestant (who was) paid the money became unavailable and suddenly terminated his contract.
    (b) After the contestant lost(,) the money became unavailable despite his previous three wins in a row.
    (c) The contestant revealed (that) the money became unavailable to him when the show's budget shrank.
23. (a) The new chef (who was) offered the restaurant separated mediocre cooks from gifted ones.
    (b) Once the new chef started(,) the restaurant separated mediocre cooks from gifted ones.
    (c) The new chef claimed (that) the restaurant separated mediocre cooks from gifted ones.
24. (a) The apprentice baker (who was) assigned the oven produced smaller cakes because he lacked experience.
    (b) When the apprentice baker worked(,) the oven produced smaller cakes because he lacked experience.
    (c) The apprentice baker discovered (that) the oven produced smaller cakes because it heated too fast.

B. Relative clause subset.

1. (a) **Object Relative Clause (ORC)**: The bus driver who the kids followed wondered about the location of a hotel.
   (b) **Subject Relative Clause (SRC)**: The bus driver who followed the kids wondered about the location of a hotel.
2. (a) The chef who the cameraman distracted poured the flour onto the counter.
   (b) The chef who distracted the cameraman poured the flour onto the counter.
3. (a) The children who the father woke bothered him about the trip to the beach.
   (b) The children who woke the father bothered him about the trip to the beach.
4. (a) The class that the teacher disliked skimmed the reading for the week.
   (b) The class that disliked the teacher skimmed the reading for the week.
5. (a) The dancer that the audience loved ignored some basic principles.
   (b) The dancer that loved the audience ignored some basic principles.
6. (a) The employees that the fireman noticed hurried across the open field.
   (b) The employees that noticed the fireman hurried across the open field.
7. (a) The farmer that the customers approached lifted the chickens from their coop.
   (b) The farmer that approached the customers lifted the chickens from their coop.
8. (a) The farmer who the rancher hired piled the seeds in long rows.
   (b) The farmer who hired the rancher piled the seeds in long rows.
9. (a) The firemen that the residents called attacked the house with high-powered hoses.
   (b) The firemen that called the residents attacked the house with high-powered hoses.
10. (a) The girl who the parents watched changed a critical part of the story.
    (b) The girl who watched the parents changed a critical part of the story.
11. (a) The investigator who the agency phoned considered Ms. Reynolds from accounting.
    (b) The investigator who phoned the agency considered Ms. Reynolds from accounting.
12. (a) The judge who the witnesses addressed noticed the defense attorneys.
    (b) The judge who addressed the witnesses noticed the defense attorneys.
13. (a) The manager who the boss visited remembered some inconvenient facts.
    (b) The manager who visited the boss remembered some inconvenient facts.
14. (a) The mathematician who the chairman visited created a solution to the well-known problem.
    (b) The mathematician who visited the chairman created a solution to the well-known problem.
15. (a) The monkeys that the zookeepers watched charged the bars of their cage.
    (b) The monkeys that watched the zookeepers charged the bars of their cage.
16. (a) The movie star who the organizers visited proposed an annual prize.
    (b) The movie star who visited the organizers proposed an annual prize.
17. (a) The neighbor who the couple observed purchased the old Victorian house.
    (b) The neighbor who observed the couple purchased the old Victorian house.
18. (a) The pilot who the ground crew delayed remained on the runway for a long time.
    (b) The pilot who delayed the ground crew remained on the runway for a long time.
19. (a) The soldiers that the natives helped climbed the big rock that blocked the path.
    (b) The soldiers that helped the natives climbed the big rock that blocked the path.
20. (a) The speaker who the economists entertained predicted a good year for the industry.
    (b) The speaker who entertained the economists predicted a good year for the industry.

21. (a) The table top that the box rested on screwed directly to the legs.
    (b) The table top that rested on the box screwed directly to the legs.
22. (a) The trainer who the jockey called rubbed the horse's skin.
    (b) The trainer who called the jockey rubbed the horse's skin.
23. (a) The veteran who the coach admired defeated his greatest rival.
    (b) The veteran who admired the coach defeated his greatest rival.
24. (a) The visitor who the student introduced walked across the quad.
    (b) The visitor who introduced the student walked across the quad.

C. Attachment ambiguity subset.

1. (a) **Low Attachment**: In the lobby, Clyde bumped into the chauffeurs of the CEO who is reckless and very unpopular with the company.
   (b) **High Attachment**: In the lobby, Clyde bumped into the chauffeur of the CEOs who is reckless and very unpopular with the company.
   (c) **Ambiguous**: In the lobby, Clyde bumped into the chauffeur of the CEO who is reckless and very unpopular with the company.
2. (a) Edwin has been reading about the sisters of the actor who was visiting the resort in Death Valley.
   (b) Edwin has been reading about the sister of the actors who was visiting the resort in Death Valley.
   (c) Edwin has been reading about the sister of the actor who was visiting the resort in Death Valley.
3. (a) From the gallery, Franny observed the nurses of the surgeon who was in charge of the operation currently underway.
   (b) From the gallery, Franny observed the nurse of the surgeons who was in charge of the operation currently underway.
   (c) From the gallery, Franny observed the nurse of the surgeon who was in charge of the operation currently underway.
4. (a) Gerald introduced himself to the nieces of the billionaire who sails vintage yachts around the Vineyard.
   (b) Gerald introduced himself to the niece of the billionaires who sails vintage yachts around the Vineyard.
   (c) Gerald introduced himself to the niece of the billionaire who sails vintage yachts around the Vineyard.
5. (a) At the potluck, Marcus chatted with the aunts of the nun who bakes sugar cookies with cute designs.
   (b) At the potluck, Marcus chatted with the aunt of the nuns who bakes sugar cookies with cute designs.
   (c) At the potluck, Marcus chatted with the aunt of the nun who bakes sugar cookies with cute designs.
6. (a) During the budget negotiation, Janet charmed the assistants of the executive who decides almost everything in secret.
   (b) During the budget negotiation, Janet charmed the assistant of the executives who decides almost everything in secret.
   (c) During the budget negotiation, Janet charmed the assistant of the executive who decides almost everything in secret.
7. (a) On the fishing trip, we laughed at the uncles of the sailor who was confused about the motor on the boat.
   (b) On the fishing trip, we laughed at the uncle of the sailors who was confused about the motor on the boat.
   (c) On the fishing trip, we laughed at the uncle of the sailor who was confused about the motor on the boat.
8. (a) At trial, we scrutinized the prisoners of the FBI agent who was lying about the incident at the casino.
   (b) At trial, we scrutinized the prisoner of the FBI agents who was lying about the incident at the casino.
   (c) At trial, we scrutinized the prisoner of the FBI agent who was lying about the incident at the casino.
9. (a) During the demonstration, someone photographed the soldiers of the lieutenant who was camouflaged and hiding in the trees.
   (b) During the demonstration, someone photographed the soldier of the lieutenants who was camouflaged and hiding in the trees.
   (c) During the demonstration, someone photographed the soldier of the lieutenant who was camouflaged and hiding in the trees.

10. (a) Karl recognized the hostages of the pirate who was on TV this morning on the local news.
    (b) Karl recognized the hostage of the pirates who was on TV this morning on the local news.
    (c) Karl recognized the hostage of the pirate who was on TV this morning on the local news.
11. (a) During the play, we all heckled the murderers of the prince who was disguised as a peasant from nearby Trosselheim.
    (b) During the play, we all heckled the murderer of the princes who was disguised as a peasant from nearby Trosselheim.
    (c) During the play, we all heckled the murderer of the prince who was disguised as a peasant from nearby Trosselheim.
12. (a) At the charity show, Noreen nodded to the sidekicks of the actor who was juggling sharp knives and glass bottles.
    (b) At the charity show, Noreen nodded to the sidekick of the actors who was juggling sharp knives and glass bottles.
    (c) At the charity show, Noreen nodded to the sidekick of the actor who was juggling sharp knives and glass bottles.
13. (a) No one quite knew how to respond to the buddy of the janitors who burp without excusing themselves.
    (b) No one quite knew how to respond to the buddies of the janitor who burp without excusing themselves.
    (c) No one quite knew how to respond to the buddies of the janitors who burp without excusing themselves.
14. (a) The cunning Wally outmaneuvered the henchman of the villains who often fail to carry out the plot.
    (b) The cunning Wally outmaneuvered the henchmen of the villain who often fail to carry out the plot.
    (c) The cunning Wally outmaneuvered the henchmen of the villains who often fail to carry out the plot.
15. (a) Down at the pub, Ollie gossiped about the daughter of the nurses who were at church last Sunday in grimy shorts.
    (b) Down at the pub, Ollie gossiped about the daughters of the nurse who were at church last Sunday in grimy shorts.
    (c) Down at the pub, Ollie gossiped about the daughters of the nurses who were at church last Sunday in grimy shorts.
16. (a) From the lounge everyone could see the pilot of the millionaires who were distrusted by everyone at the company.
    (b) From the lounge everyone could see the pilots of the millionaire who were distrusted by everyone at the company.
    (c) From the lounge everyone could see the pilots of the millionaires who were distrusted by everyone at the company.
17. (a) On the news they showed the accomplice of the thieves who were indicted for stealing the Mona Lisa.
    (b) On the news they showed the accomplices of the thief who were indicted for stealing the Mona Lisa.
    (c) On the news they showed the accomplices of the thieves who were indicted for stealing the Mona Lisa.
18. (a) Everyone at the party groaned at the bodyguard of the divas who smoke clove cigarettes constantly.
    (b) Everyone at the party groaned at the bodyguards of the diva who smoke clove cigarettes constantly.
    (c) Everyone at the party groaned at the bodyguards of the divas who smoke clove cigarettes constantly.
19. (a) At the summit, Ursula warmly greeted the advisor of the tycoons who snowboard in Aspen in January.
    (b) At the summit, Ursula warmly greeted the advisors of the tycoon who snowboard in Aspen in January.
    (c) At the summit, Ursula warmly greeted the advisors of the tycoons who snowboard in Aspen in January.
20. (a) Rosalina testified against the detective of the senators who were caught spying on his colleagues.
    (b) Rosalina testified against the detectives of the senator who were caught spying on his colleagues.
    (c) Rosalina testified against the detectives of the senators who were caught spying on his colleagues.
21. (a) Before the exhibition, Silas telephoned the friend of the bodybuilders who write fan fiction about Batman.
    (b) Before the exhibition, Silas telephoned the friends of the bodybuilder who write fan fiction about Batman.
    (c) Before the exhibition, Silas telephoned the friends of the bodybuilders who write fan fiction about Batman.
22. (a) At her orientation, Tamara recently met the nephew of the professors who paint beautiful portraits of local celebrities.
    (b) At her orientation, Tamara recently met the nephews of the professor who paint beautiful portraits of local celebrities.
    (c) At her orientation, Tamara recently met the nephews of the professors who paint beautiful portraits of local celebrities.
23. (a) Everyone at the coffee shop sympathized with the courier of the florists who were complaining about the weather.
    (b) Everyone at the coffee shop sympathized with the couriers of the florist who were complaining about the weather.
    (c) Everyone at the coffee shop sympathized with the couriers of the florists who were complaining about the weather.

24. (a) Despite the good press, we didn't really like the commander of the soldiers who whistle very loudly and for no reason at all.
    (b) Despite the good press, we didn't really like the commanders of the soldier who whistle very loudly and for no reason at all.
    (c) Despite the good press, we didn't really like the commanders of the soldiers who whistle very loudly and for no reason at all.

D. Agreement violation subset.

1. (a) **Violation**: If the supervisor changes, the schedules deserves further inspection by the rest of the staff.
   (b) **No Violation**: If the supervisor changes, the schedule deserves further inspection by the rest of the staff.
2. (a) When the magician moves, the cards disappears mysteriously from his assistant's hand.
   (b) When the magician moves, the card disappears mysteriously from his assistant's hand.
3. (a) Whenever the lawyer leaves, his clients appears increasingly uncomfortable in the courtroom.
   (b) Whenever the lawyer leaves, his client appears increasingly uncomfortable in the courtroom.
4. (a) After the esteemed reviewer reads, the books gains more attention due to his glowing praise.
   (b) After the esteemed reviewer reads, the book gains more attention due to his glowing praise.
5. (a) Whenever the nurse calls, the doctors stops working immediately to check on the patient.
   (b) Whenever the nurse calls, the doctor stops working immediately to check on the patient.
6. (a) When the lecturer stops, her audiences needs several minutes to reflect on the content.
   (b) When the lecturer stops, her audience needs several minutes to reflect on the content.
7. (a) When the actress begins, the scenes fails dramatically despite the months she spent rehearsing.
   (b) When the actress begins, the scene fails dramatically despite the months she spent rehearsing.
8. (a) After the worst team loses, the tournaments remains essentially the same for the rest of the year.
   (b) After the worst team loses, the tournament remains essentially the same for the rest of the year.
9. (a) When the supervisor works, the shifts seems unnecessarily stressful on a Friday night.
   (b) When the supervisor works, the shift seems unnecessarily stressful on a Friday night.
10. (a) After the diplomat signs, the agreements creates another border conflict as a side effect.
    (b) After the diplomat signs, the agreement creates another border conflict as a side effect.
11. (a) Whenever the reporter moves, the cameras requires constant adjustment from the director.
    (b) Whenever the reporter moves, the camera requires constant adjustment from the director.
12. (a) Unless the dog attacks, the cats remains relatively tranquil throughout the day.
    (b) Unless the dog attacks, the cat remains relatively tranquil throughout the day.
13. (a) Until the lead architect leaves, the projects demands immense patience from the engineers.
    (b) Until the lead architect leaves, the project demands immense patience from the engineers.
14. (a) Even if the mother calls, her boys continues causing problems with the other kids on the playground.
    (b) Even if the mother calls, her boy continues causing problems with the other kids on the playground.
15. (a) After the tutor stops, the students starts working independently on the questions.
    (b) After the tutor stops, the student starts working independently on the questions.
16. (a) Once the head surgeon begins, the operations achieves incredible results given the risks involved.
    (b) Once the head surgeon begins, the operation achieves incredible results given the risks involved.
17. (a) After the producer starts, the auditions separates mediocre actors from talented ones.
    (b) After the producer starts, the audition separates mediocre actors from talented ones.
18. (a) However hard the scientist works, his experiments produces smaller amounts of alcohol than expected.
    (b) However hard the scientist works, his experiment produces smaller amounts of alcohol than expected.

E. Filler items.

1. There are now rumblings that Apple might soon invade the smart watch space, though the company is maintaining its customary silence.
2. A bill was drafted and introduced into Parliament several times but met with great opposition, mostly from farmers.
3. The human body can tolerate only a small range of temperature, especially when the person is engaged in vigorous activity.
4. Seeing Peter slowly advancing upon him through the air with dagger poised, he sprang upon the bulwarks to cast himself into the sea.
5. Some months later, Michael Larson saw another opportunity to stack the odds in his favor with a dash of ingenuity.
6. Bob Murphy, the Senior PGA Tour money leader with seven hundred thousand, says heat shouldn't be a factor.
7. Greg Anderson, considered a key witness by the prosecution, vowed he wouldn't testify when served a subpoena last week.
8. Owls are more flexible than humans because a bird's head is only connected by one socket pivot.
9. Even in the same animal, not all bites are the same.
10. Buck did not like it, but he bore up well to the work, taking pride in it.
11. These days, neuroscience is beginning to catch up to musicians who practice mentally.
12. Hybrid vehicles have a halo that makes owners feel righteous and their neighbors feel guilty for not doing as much to save the planet.
13. Binge drinking may not necessarily kill or even damage brain cells, as commonly thought, a new animal study suggests.
14. When attacked, a skunk's natural inclination is to turn around, lick its tail and spray a noxious scent.
15. All that the brain has to work with are imperfect incoming electrical impulses announcing that things are happening.
16. There often seems to be more diving in soccer than in the Summer Olympics.
17. Susan B. Anthony spent nearly sixty years of her life devoted to the cause of social justice and equality for all.
18. Unfortunately, for every six water bottles we use, only one makes it to the recycling bin.
19. As in the United States, Colombian legislation requires travelers entering the country to declare cash in excess of ten thousand dollars.
20. Stress is a risk factor for both depression and anxiety, he says.
21. When it comes to having a lasting and fulfilling relationship, common wisdom says that feeling close to your romantic partner is paramount.
22. Voltaire himself probably won around half a million livres, a large fortune, which he then made even larger.
23. When preparing to check out of their hotel room, some frequent travelers pile up their used bath towels on the bathroom floor.
24. Steam sterilization is limited in the types of medical waste it can treat, but is appropriate for laboratory substances contaminated with infectious organisms.
25. When the new world was first discovered it was found to be, like the old, well stocked with plants and animals.
26. Police in Georgia have shut down a lemonade stand run by three girls trying to save up for a trip to a water park.
27. An early task will be to make sure the newfound microbes were not introduced while drilling through the ice into the lake.
28. Lady Gaga's YouTube account was suspended Thursday.
29. John Thornton asked little of man or nature.
30. Proper ventilation will make a backdraft less likely.
31. For centuries, time was measured by the position of the sun with the use of sundials.

32. The girl's feet were then re-wrapped even tighter than before, causing her footprint to shrink further.

33. The astronauts used a hefty robotic arm to move the bus-size canister, stuffed with nearly three tons of packing foam.

34. Very similar, but even more striking, is the evidence from athletic training.

35. I agree that California's "three strikes and you're out" law will be a financial disaster for taxpayers who care about education.

36. It was a forbidding challenge, and it says much for Winstanley's persuasive abilities, not to mention his self-confidence.

37. With schools still closed, cars still buried and streets still blocked by the widespread weekend snowstorm, officials are asking people to help out.

38. Research showing that a tiny European river bug called the water boatman may be the loudest animal on earth.

39. From coal to cars, Chinese floods tangle supply chains worldwide.

40. This new film marks 10 years since the death of the superstar.

References

1. BD Oh, W Schuler, Leading Whitespaces of Language Models' Subword Vocabulary Pose a Confound for Calculating Word Probabilities in *Proceedings of the 2024 Conference on Empirical Methods in Natural Language Processing*, eds. Y Al-Onaizan, M Bansal, YN Chen. (Association for Computational Linguistics, Miami, Florida, USA), pp. 3464–3472 (2024).
2. T Pimentel, C Meister, How to Compute the Probability of a Word in *Proceedings of the 2024 Conference on Empirical Methods in Natural Language Processing*, eds. Y Al-Onaizan, M Bansal, YN Chen. (Association for Computational Linguistics, Miami, Florida, USA), pp. 18358–18375 (2024).
3. S Biderman, et al., Pythia: A suite for analyzing large language models across training and scaling in *Proceedings of the 40th International Conference on Machine Learning.* Vol. 202, pp. 2397–2430 (2023).
4. A Vaswani, et al., Attention is all you need in *Advances in Neural Information Processing Systems.* Vol. 30, pp. 6000–6010 (2017).
5. L Gao, et al., The Pile: An 800GB dataset of diverse text for language modeling. *arXiv preprint* **arXiv:2101.00027** (2020).
6. A Radford, et al., Language models are unsupervised multitask learners. *OpenAI Tech. Rep.* (2019).
7. A Gu, T Dao, Mamba: Linear-time sequence modeling with selective state spaces. *arXiv preprint* **arXiv:2312.00752v2** (2023).
8. M van Schijndel, A Mueller, T Linzen, Quantity doesn't buy quality syntax with neural language models in *Proceedings of the 2019 Conference on Empirical Methods in Natural Language Processing and the 9th International Joint Conference on Natural Language Processing (EMNLP-IJCNLP).* pp. 5831–5837 (2019).
9. S Hochreiter, J Schmidhuber, Long short-term memory. *Neural Comput.* **9**, 1735–1780 (1997).
10. S Merity, C Xiong, J Bradbury, R Socher, Pointer sentinel mixture models. *arXiv preprint* **arXiv:1609.07843** (2016).
11. C Dyer, A Kuncoro, M Ballesteros, NA Smith, Recurrent neural network grammars in *Proceedings of the 2016 Conference of the North American Chapter of the Association for Computational Linguistics: Human Language Technologies*. pp. 199–209 (2016).
12. H Noji, Y Oseki, Effective batching for recurrent neural network grammars in *Findings of the Association for Computational Linguistics: ACL-IJCNLP 2021.* pp. 4340–4352 (2021).
13. E Charniak, et al., BLLIP 1987-89 WSJ Corpus Release 1 (2000).
14. M Stern, D Fried, D Klein, Effective inference for generative neural parsing in *Proceedings of the 2017 Conference on Empirical Methods in Natural Language Processing.* pp. 1695–1700 (2017).
15. K Gulordava, P Bojanowski, E Grave, T Linzen, M Baroni, Colorless green recurrent networks dream hierarchically in *Proceedings of the 2018 Conference of the North American Chapter of the Association for Computational Linguistics: Human Language Technologies.* pp. 1195–1205 (2018).
16. M Hahn, R Futrell, R Levy, E Gibson, A resource-rational model of human processing of recursive linguistic structure. *Proc. Natl. Acad. Sci.* **119**, e2122602119 (2022).
17. K McCurdy, M Hahn, Lossy Context Surprisal Predicts Task-Dependent Patterns in Relative Clause Processing in *Proceedings of the 28th Conference on Computational Natural Language Learning*, eds. L Barak, M Alikhani. (Association for Computational Linguistics, Miami, FL, USA), pp. 36–45 (2024).
18. R Futrell, et al., Neural language models as psycholinguistic subjects: Representations of syntactic state in *Proceedings of the 2019 Conference of the North American Chapter of the Association for Computational Linguistics: Human Language Technologies.* pp. 32–42 (2019).
19. GTM Altmann, A Garnham, Y Dennis, Avoiding the garden path: Eye movements in context. *J. Mem. Lang.* **31**, 685–712 (1992).

20. K Rayner, SC Sereno, Regressive eye movements and sentence parsing: On the use of regression-contingent analyses. *Mem. & Cogn.* **22**, 281–285 (1994).
21. GTM Altmann, Regression-contingent analyses of eye movements during sentence processing: Reply to Rayner and Sereno. *Mem. & Cogn.* **22**, 286–290 (1994).
22. W Vonk, R Cozijn, Chapter 15 - On the treatment of saccades and regressions in eye movement measures of reading time in *The Mind's Eye*, eds. J Hyönä, R Radach, H Deubel. (North-Holland), pp. 291–311 (2003).
23. ER Schotter, B Dillon, A beginner's guide to eye tracking for psycholinguistic studies of reading. *Behav. Res. Methods* **57**, 68 (2025).
24. ED Reichle, T Warren, K McConnell, Using E-Z Reader to model the effects of higher level language processing on eye movements during reading. *Psychon. Bull. & Rev.* **16**, 1–21 (2009).
25. C Clifton Jr., A Staub, K Rayner, Chapter 15 - Eye movements in reading words and sentences in *Eye Movements: A Window on Mind and Brain*, eds. RPG van Gompel, MH Fischer, WS Murray, RL Hill. (Elsevier), pp. 341–371 (2007).
26. A Staub, Word recognition and syntactic attachment in reading: Evidence for a staged architecture. *J. Exp. Psychol. Gen.* **140**, 407–433 (2011).
27. A Staub, Eye movements and processing difficulty in object relative clauses. *Cognition* **116**, 71–86 (2010).
28. SL Frank, R Bod, Insensitivity of the Human Sentence-Processing System to Hierarchical Structure. *Psychol. Sci.* **22**, 829–834 (2011).
29. A Goodkind, K Bicknell, Predictive power of word surprisal for reading times is a linear function of language model quality in *Proceedings of the 8th Workshop on Cognitive Modeling and Computational Linguistics (CMCL 2018)*, eds. A Sayeed, C Jacobs, T Linzen, M van Schijndel. (Association for Computational Linguistics, Salt Lake City, Utah), pp. 10–18 (2018).
30. EG Wilcox, J Gauthier, J Hu, P Qian, RP Levy, On the predictive power of neural language models for human real-time comprehension behavior in *Proceedings of the Annual Meeting of the Cognitive Science Society*. Vol. 42, pp. 1707–1713 (2020).
31. M Schrimpf, et al., The neural architecture of language: Integrative modeling converges on predictive processing. *Proc. Natl. Acad. Sci.* **118**, e2105646118 (2021).
32. KJ Huang, et al., Large-scale benchmark yields no evidence that language model surprisal explains syntactic disambiguation difficulty. *J. Mem. Lang.* **137**, 104510 (2024).
33. CR Gallistel, The importance of proving the null. *Psychol. Rev.* **116**, 439–453 (2009).
34. JN Rouder, PL Speckman, D Sun, RD Morey, G Iverson, Bayesian t tests for accepting and rejecting the null hypothesis. *Psychon. Bull. & Rev.* **16**, 225–237 (2009).
35. S Chinn, A simple method for converting an odds ratio to effect size for use in meta-analysis. *Stat. Medicine* **19**, 3127–3131 (2000).
36. MD Lee, EJ Wagenmakers, *Bayesian Cognitive Modeling: A Practical Course*. (Cambridge University Press, Cambridge), (2014).
37. PC Bürkner, brms: An R Package for Bayesian multilevel models using Stan. *J. Stat. Softw.* **80**, 1–28 (2017).
38. M van Schijndel, T Linzen, Single-stage prediction models do not explain the magnitude of syntactic disambiguation difficulty. *Cogn. Sci.* **45**, e12988 (2021).
39. S Arehalli, B Dillon, T Linzen, Syntactic surprisal from neural models predicts, but underestimates, human processing difficulty from syntactic ambiguities in *Proceedings of the 26th Conference on Computational Natural Language Learning*. pp. 301–313 (2022).
40. BD Oh, W Schuler, Transformer-based language model surprisal predicts human reading times best with about two billion training tokens in *Findings of the Association for Computational Linguistics: EMNLP 2023*. pp. 1915–1921 (2023).
41. BD Oh, W Schuler, Why does surprisal from larger Transformer-based language models provide a poorer fit to human reading times? *Transactions Assoc. for Comput. Linguist.* **11**, 336–350 (2023).
42. BD Oh, S Yue, W Schuler, Frequency explains the inverse correlation of large language models' size, training data amount, and surprisal's fit to reading times in *Proceedings of the 18th Conference of the European Chapter of the Association for Computational Linguistics*. pp. 2644–2663 (2024).
43. EG Wilcox, et al., Bigger is not always better: The importance of human-scale language modeling for psycholinguistics. *J. Mem. Lang.* **144**, 104650 (2025).
44. BD Oh, T Linzen, To model human linguistic prediction, make LLMs less superhuman. *arXiv preprint* **arXiv:2510.05141v1** (2025).
